# Diversity of xerotolerant and xerophilic fungi in honey

**DOI:** 10.1186/s43008-019-0021-7

**Published:** 2019-11-27

**Authors:** E. Rodríguez-Andrade, A. M. Stchigel, A. Terrab, J. Guarro, J. F. Cano-Lira

**Affiliations:** 1 Mycology Unit, Medical School and IISPV, Universitat Rovira i Virgili (URV), Sant Llorenç 21, 43201 Reus, Tarragona, Spain; 2 Department of Plant Biology and Ecology, University of Seville, 41012 Seville, Spain

**Keywords:** *Eurotiales*, Fungi, Honey, New taxa, *Onygenales*, Osmophiles, Xerophiles

## Abstract

Fungi can colonize most of the substrata on Earth. Honey, a sugary food produced by bees (and other insects) has been studied little in terms of its fungal diversity. We have surveyed and evaluated the presence of xerotolerant and xerophilic fungi in a set of honey bee samples collected from across Spain. From 84 samples, a total of 104 fungal strains were isolated, and morphologically and phylogenetically characterized. We identified 32 species distributed across 16 genera, most of them belonging to the ascomycetous genera *Aspergillus*, *Bettsia*, *Candida*, *Eremascus*, *Monascus*, *Oidiodendron*, *Penicillium*, *Skoua*, *Talaromyces* and *Zygosaccharomyces*. As a result of this survey, eight new taxa are proposed: i.e. the new family *Helicoarthrosporaceae,* two new genera, *Helicoarthrosporum* and *Strongyloarthrosporum* in *Onygenales*; three new species of *Eurotiales*, *Talaromyces affinitatimellis*, *T. basipetosporus*, and *T. brunneosporus*; and two new species of *Myxotrichaceae, Oidiodendron mellicola,* and *Skoua asexualis*.

## INTRODUCTION

Honey is a natural sweetener produced by honey bees (insects of the genus *Apis* of the order *Hymenoptera*) from nectar (blossom honey or nectar honey) or from carbohydrate-rich secretions of living green parts of plants or excretions of plant-sucking phytophagous aphids (insects of the family *Aphidida*, order *Hemiptera*) (honeydew honey) after combination with the bee’s specific substances, placement, dehydration, and storage in the honey comb to ripen and mature. Honey is mostly composed of monosaccharides (dextrose and fructose), at a concentration of not lower than 60% and a much lesser amount of oligosaccharides, organic acids, enzymes (amylases and α-glucosidase) and solid particles. Due to its particular physicochemical nature and biological origin, honey should be an ideal substratum for the development of xerotolerant and xerophilic fungi. However, little information has been gathered about these fungi and their relationships with honey and honey products. Nonetheless, most of the fungal species from honey had been reported as new for science. Representative ascomycetous yeasts found in honey are *Blastobotrys meliponae*, *Candida lundiana*, *C. magnoliae*, *C. sorbosivorans*, *C. suthepensis*, *Schizosaccharomyces octosporus*, *Trichosporon mucoides*, *Zygosaccharomyces favi*, *Z. mellis*, *Z. richteri*, *Z. rouxii,* and *Z. siamensis* (Lochhead & Farrell 1931; Ruiz-Argueso & Rodriguez-Navarro 1975; Carvalho et al. 2010; Saksinchai et al. 2012a, b; Čadež et al. 2015; Crous et al. 2016). The obligate xerophiles *Ascosphaera apis* and *Bettsia alvei* have been reported in honey, as well as several xerotolerant species of *Alternaria*, *Aspergillus*, *Cladosporium* and *Penicillium* and a few mucoralean fungi (Snowdon & Cliver 1996; Kačániová et al. 2009; Pettersson & Leong 2011; Kačániová et al. 2012; Sinacori et al. 2014; Grabowski & Klein 2015). Recently, *Monascus mellicola, Penicillium apimei*, *P. meliponae*, *P. mellis,* and *Talaromyces brasiliensis* were reported from honey produced by stingless bees (*Melipona scutellaris*, family *Apidae*, order *Hymenoptera*) inhabiting Brazilian forests (Barbosa et al. 2017, 2018). Common environmental and plant pathogenic species of fungi have been reported in samples of honey collected in Spain (Pérez-Sánchez et al. 1997; Seijo et al. 2011; Magyar et al. 2016; Terrab et al. 2019) and Portugal (Martíns et al. 2003). In another study, the yeast *Metschnikowia reukaufii* was, surprisingly, the only fungus reported for floral honey from Portugal and Spain (Magyar et al. 2005). Although honey should be a substratum amenable for the development of xerotolerant and xerophilic fungi, few studies have intentionally targeted these fungi. Therefore, the main objective of this study was to assess the diversity of honey-associated fungi, by employing a selective culture medium to a set of samples collected predominantly in Spain, and to characterize the morphology, physiology and phylogeny of new isolates and those considered of taxonomic interest.

## MATERIALS AND METHODS

### Fungal isolation

**Fig. 1:**
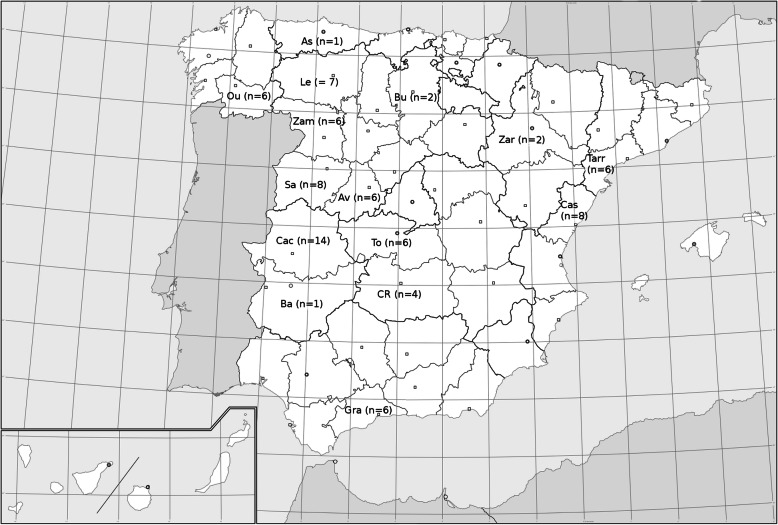
Locations of Spain where the samples were collected. Asturias (As), Ávila (Av), Badajoz (Ba), Burgos (Bu), Cáceres (Cac), Castellón (Cas), Ciudad Real (CR), Granada (Gra), León (Le), Ourense (Ou), Salamanca (Sa), Tarragona (Tarr), Toledo (To), Zamora (Zam), and Zaragoza (Zar)

A total of 83 samples of honeydew and blossom (nectar) honey from different locations in Spain (Fig. 1), and one from Argentina (San Martín, Buenos Aires province), have been processed. All samples were of the harvest in 2014, stored in settling tanks, and after a variable period of time clarified by filtration (with one exception, which was by centrifugation). Seventy-two of the Spanish samples corresponded to honeydew honeys, 45 from trading companies and 27 collected and processed by beekeepers. A few of the samples provided by commercial companies were categorized (according to the nature of the honeydew) as oak, holm oak and forest honey. The 11 samples of blossom honey were provided by beekeepers, and these were classified as multifloral. All samples provided by commercial companies were subjected to a thermal treatment, subjecting the honey at 45–55 °C for a few hours up to 2 days, or pasteurized (2 min at 80 °C). The samples provided by beekeepers have not undergone any heat treatment. For each sample, 10 g of honey was dissolved into 90 mL of sterile water in a sterile disposable plastic container, and 1 mL of such dilution (1:10) was aseptically plated onto two 90 mm diam. plastic Petri dishes and mixed with 15 mL of molten (at 50–55 °C) 18% glycerol agar (G18; DG18 [Hocking & Pitt 1980] without dichloran: 5 g peptone, 10 g dextrose, 1 g KH_2_PO_4_, 0.5 g MgSO_4_·7H_2_O, 15 g agar-agar, 110 g glycerol, 1 L tap water, and supplemented with 250 mg/L of L-chloramphenicol). Once the medium had solidified, one of the Petri dishes was incubated in darkness at 15 °C and the other at 25 °C for up to 2 months. The colonies developed were examined under a stereomicroscope. Fungal structures from selected (representative of all morphological variety) colonies were transferred to 50 mm diam. Petri dishes containing G18 by using a sterile insulin-type needle and incubated in the same conditions to obtain pure cultures.

### Phenotypic study

For cultural characterization, suspensions of spores from the isolates were prepared in a semi-solid medium (0.2% agar; 0.05% Tween 80), and 0.5 μL of such suspension was inoculated onto malt extract agar (MEA; Difco, Detroit, USA; Samson et al. 2010), oatmeal agar (OA; 30 g of filtered oat flakes, 15 g agar-agar, 1 L tap water; Samson et al. 2010), Czapek yeast extract agar (CYA; 30 g sucrose, 3 g NaNO_3_, 5 g yeast extract, 1 g K_2_HPO_4_, 0.5 g KCl, 0.5 g MgSO_4_·7H_2_O, 0.01 g FeSO_4_, 15 g agar-agar, 1 L tap water; Pitt 1979), yeast extract sucrose agar (YES; 20 g yeast extract, 150 g sucrose, 0.5 g MgSO_4_·7H_2_O, 20 g agar-agar, 1 L tap water; Frisvad 1981), creatine sucrose agar (CREA; 3 g creatine, 30 g sucrose, 1.6 g K_3_PO_4_·7H_2_O, 0.5 g MgSO_4_·7H_2_O, 0.5 g KCl, 0.01 g FeSO_4_·7H_2_O, 0.05 g bromocresol purple, 20 g agar-agar, 1 L tap water; Frisvad 1981), G18, potato dextrose agar (PDA; Pronadisa, Madrid, Spain; Hawksworth et al. 1995), 25% glycerol nitrate agar (G25 N; 7.5 g Czapek concentrate, 0.75 g K_2_HPO_4_, 3.7 g yeast extract, 250 mL glycerol, 12 g agar-agar, 1 L tap water; Pitt 1979), bromocresol purple milk solids glucose agar (BCP-MS-G; 80 g skim milk powder, 40 g glucose, 10 mL of 1.6% of bromocresol purple in 95% ethanol, 30 g agar-agar,1 L tap water; Kane & Smitka 1978), test opacity tween medium (TOTM; 10 g bacteriological peptone, 5 g NaCl, 1 g CaCl_2_, 5 mL Tween 80, 15 g agar-agar, 1 L tap water; Slifkin 2000), phytone yeast extract agar (PYE; Becton, Dickinson & Co., Sparks, MD, USA; Carmichael & Kraus 1959), malt extract yeast extract 70% fructose-glucose (MY70FG; 6 g malt extract, 6 g yeast extract, 10 g peptone, 350 g fructose, 350 g glucose, 12 g agar-agar, 1 L tap water; Beuchat & Hocking 1990), and blood agar (Becton, Dickinson & Co., Sparks, MD, USA). Colonies were characterized after three wk. at 25 °C in darkness. G18 medium was used to determine the minimum, optimal and maximum temperatures of growth. Christensen’s urea agar (EMD Millipore, Darmstadt, Germany; Christensen 1946) was inoculated and incubated during 4–7 days at 25 °C in darkness to detect the production of urease. Cycloheximide tolerance of the fungal strains was tested on Sabouraud dextrose agar (SDA; Pronadisa, Spain) supplemented with 0.2% of cycloheximide (Sigma, USA) after incubation at 30 °C for two wk. Fungal tolerance to NaCl was evaluated on SDA adding 3, 10 and 20% w/v NaCl, with the same incubation conditions as in the previous test. Colour notations were according to Kornerup & Wanscher (1978). The microscopic structures were characterized and measured from wet mountings of slide cultures, using water and 60% lactic acid. Photo micrographs were taken using a Zeiss Axio-Imager M1 light microscope (Oberkochen, Germany) with a DeltaPix Infinity X digital camera, using Nomarski differential interference contrast. The samples for scanning electron microscopy (SEM) were processed according to Figueras & Guarro (1988), and SEM micrographs were taken at 15 keV with a JEOL JSM 840 microscope.

### DNA extraction, amplification and sequencing

Total deoxyribonucleic acid (DNA) was extracted according to Marimon et al. (2006), and a fragment of the 28S nrRNA gene (LSU) was amplified and sequenced using the primer pair LR0R (Rehner & Samuels 1994) and LR5 (Vilgalys & Hester 1990). For some isolates the following markers were amplified and sequenced: ribosomal internal transcribed spacers (ITS) (ITS5/ITS4; White et al. 1990); and fragments of the beta-tubulin (*BenA*) (Bt2a/Bt2b; Glass & Donaldson 1995), calmodulin (*CaM*) (Cmd5/Cmd6; Hong et al. 2005) and RNA polymerase II subunit 2 (*rpb*2) (RPB2-5F/RPB2-7cR; Liu et al. 1999) genes. Amplicons were sequenced at Macrogen Europe (Macrogen, Amsterdam, The Netherlands). Consensus sequences were obtained using the SeqMan software v. 7 (DNAStar Lasergene, Madison, WI, USA). Sequences we generated were deposited in GenBank (Table 1).

**Table 1:** Fungal taxa recovered with their nucleotide sequence accession number, and the geographic origin of the honey samples processed

Taxon	Culture collection accession number	EMBL/GenBank nucleotide sequence accession number					Geographic origin (province, community)
		*BenA*	*CaM*	*rpb*2	ITS	LSU	
*Alternaria multiformis*	FMR 16018	–	–	–	LT963545	LT963546	Salamanca, Castilla y León
*Ascosphaera atra*	FMR 16318	–	–	–	LT964944	LT984552	Cáceres, Extremadura
*Aspergillus asperescens*	FMR 16310	LT963510	–	–		LT986672	Zamora, Castilla y León
*Aspergillus montevidensis*	FMR 15994	LR027804	–	–	LT963466	LT984537	Castellón, Valencia
*Aspergillus pseudoglaucus*	FMR 9392	LT963512	–	–	–	LT984695	Castellón, Valencia
*Aspergillus pseudoglaucus*	FMR 15992	LT963513	–	–	–	LT984696	Castellón, Valencia
*Aspergillus pseudoglaucus*	FMR 15993	LT963514	–	–	–	LT984697	Castellón, Valencia
*Aspergillus pseudoglaucus*	FMR 16011	LT963518	–	–	–	LT984701	Salamanca, Castilla y León
*Aspergillus pseudoglaucus*	FMR 16112	LT963515	–	–	–	LT984698	Ciudad Real, Castilla-La Mancha
*Aspergillus pseudoglaucus*	FMR 16281	LT963516	–	–	–	LT984699	Ciudad Real, Castilla-La Mancha
*Aspergillus pseudoglaucus*	FMR 16317	LT963517	–	–	–	LT984700	Zamora, Castilla y León
*Bettsia alvei*	FMR 15670	–	–	–	–	LT963566	Castellón, Valencia
*Bettsia alvei*	FMR 15672	–	–	–	–	LT963567	Castellón, Valencia
*Bettsia alvei*	FMR 15678	–	–	–	–	LT963568	Castellón, Valencia
*Bettsia alvei*	FMR 15681	–	–	–	–	LT963569	Castellón, Valencia
*Bettsia alvei*	FMR 15685	–	–	–	–	LT963570	Castellón, Valencia
*Bettsia alvei*	FMR 16111	–	–	–	–	LT963571	Cáceres, Extremadura
*Bettsia alvei*	FMR 16115	–	–	–	–	LT963572	Toledo, Castilla-La Mancha
*Bettsia alvei*	FMR 16305	–	–	–	–	LT963574	Ourense, Galicia
*Bettsia alvei*	FMR 16313	–	–	–	–	LT963575	Ourense, Galicia
*Bettsia alvei*	FMR 16568	–	–	–	–	LT963573	Cáceres, Extremadura
*Bettsia alvei*	FMR 16570	–	–	–	–	LT963576	Ourense, Galicia
*Candida magnoliae*	FMR 16311	–	–	–	–	LT963487	Ourense, Galicia
*Candida magnoliae*	FMR 16314	–	–	–	–	LT963488	Ourense, Galicia
*Candida magnoliae*	FMR 16496	–	–	–	–	LT963486	Ourense, Galicia
*Candida sorbosivorans*	FMR 16278	–	–	–	–	LT963489	Ourense, Galicia
*Cunninghamella bertholletiae*	FMR 16008	–	–	–	LT963490	LR215930	Salamanca, Castilla y León
*Eremascus albus*	FMR 16116	–	–	–	–	LT964975	Cáceres, Extremadura
*Eremascus albus*	FMR 16118	–	–	–	–	LT964976	Cáceres, Extremadura
*Eremascus albus*	FMR 16119	–	–	–	–	LT964977	Toledo, Castilla-La Mancha
*Eremascus albus*	FMR 16493	–	–	–	–	LT964978	Cáceres, Extremadura
*Helicoarthrosporum mellicola*	FMR 15673	–	–	–	–	LT978462	Castellón, Valencia
*Helicoarthrosporum mellicola* ^T^	FMR 15679 = CBS 143838	–	–	–	–	LT906535	Castellón, Valencia
*Helicoarthrosporum mellicola*	FMR 16307	–	–	–	–	LT978463	León, castilla y León
*Helicoarthrosporum mellicola*	FMR 16308	–	–	–	–	LT906536	Zamora, Castilla y León
*Helicoarthrosporum mellicola*	FMR 16315	–	–	–	–	LT906537	Cáceres, Extremadura
*Monascus pilosus*	FMR 16306	–	–	–	LT963491	LT984551	Zamora, Castilla y León
*Monascus purpureus*	FMR 16283	–	–	–	LT963492	LR215932	Ávila, Castilla y León
*Monascus purpureus*	FMR 16316	–	–	–	LT963493	LT984550	Cáceres, Extremadura
*Monascus purpureus*	FMR 16321	–	–	–	LT963494	LR215933	Cáceres, Extremadura
*Monascus ruber*	FMR 16284	–	–	–	LT963495	LT986673	Zamora, Castilla y León
*Mucor plumbeus*	FMR 16012	–	–	–	LT963539	LR215934	Ciudad Real, Castilla-La mancha
*Mucor plumbeus*	FMR 16013	–	–	–	LT963540	LT984540	Salamanca, Castilla y León
*Mucor plumbeus*	FMR 16017	–	–	–	LT963541	LT984548	Salamanca, Castilla y León
*Oidiodendron mellicola*	FMR 15680	–	–	–	LT906540	LT978465	Tarragona, Catalonia
*Oidiodendron mellicola* ^T^	FMR 15683 = CBS 143839	–	–	–	LT906544	LT978464	Castellón, Valencia
*Oidiodendron mellicola*	FMR 16023	–	–	–	LT978506	LT978470	Salamanca, Castilla y León
*Oidiodendron mellicola*	FMR 16031	–	–	–	LT906541	LT978466	Ciudad Real, Castilla-La mancha
*Oidiodendron mellicola*	FMR 16117	–	–	–	LT978503	LT978467	Ciudad Real, Castilla-La Mancha
*Oidiodendron mellicola*	FMR 16120	–	–	–	LT978507	LT978471	Toledo, Castilla-La Mancha
*Oidiodendron mellicola*	FMR 16274	–	–	–	LT978509	LT978473	Burgos, Castilla y León
*Oidiodendron mellicola*	FMR 16282	–	–	–	LT978508	LT978472	Toledo, Castilla-La Mancha
*Oidiodendron mellicola*	FMR 16503	–	–	–	LT978504	LT978468	Ciudad Real, Castilla-La Mancha
*Oidiodendron mellicola*	FMR 16504	–	–	–	LT978505	LT978469	Ourense, Galicia
*Penicillium camemberti*	FMR 16016	LR027805	–	–	LT963578	LT984541	Salamanca, Castilla y León
*Penicillium citrinum*	FMR 16028	LT963451	–	–	–	LT984702	Salamanca, Castilla y León
*Penicillium corylophilum*	FMR 16010	LR027808	–	–	LT963581	LT984538	Asturias
*Penicillium corylophilum*	FMR 16027	LT963452	–	–	–	LT986674	Asturias
*Penicillium corylophilum*	FMR 16030	LR027809	–	–	LT963582	LT984547	Cáceres, Extremadura
*Penicillium cravenianum*	FMR 16019	LR027807	–	–	LT963580	LT984542	Salamanca, Castilla y León
*Penicillium cravenianum*	FMR 16020	LR027806	–	–	LT963579	LT984549	Cáceres, Extremadura
*Rhizopus oryzae*	FMR 16022	–	–	–	LT963543	LR215931	Cáceres, Extremadura
*Schizosaccharomyces octosporus*	FMR 16279	–	–	–	–	LT963544	Ourense, Galicia
*Skoua asexualis*	FMR 16497	–	–	–	LT964664	LT964665	Cáceres, Extremadura
*Skoua asexualis*	FMR 16567	–	–	–	LT964666	LT964667	Cáceres, Extremadura
*Skoua asexualis* ^T^	FMR 16572 = CBS 144072	–	–	–	LT964668	LT964669	León, castilla y León
*Skoua fertilis*	FMR 10812	LR585993	–	LR586005	LR585979	LT965019	Castellón, Valencia
*Skoua fertilis*	FMR 10813	LR585994	–	LR586006	LR585980	LT965023	Castellón, Valencia
*Skoua fertilis*	FMR 10814	LR585995	–	–	LR585981	LT965016	Castellón, Valencia
*Skoua fertilis*	FMR 10815	–	–	LR586007	LR585982	LT965015	Castellón, Valencia
*Skoua fertilis*	FMR 15671	LR585996	–	LR586008	LR585983	LT965014	Castellón, Valencia
*Skoua fertilis*	FMR 15676	LR585997	–	LR586009	LR585984	LT965017	Castellón, Valencia
*Skoua fertilis*	FMR 15682	LR585998	–	LR586010	LR585985	LT965018	Castellón, Valencia
*Skoua fertilis*	FMR 15686	LR585999	–	LR586011	LR585986	LT965020	Castellón, Valencia
*Skoua fertilis*	FMR 15687	LR586000	–	LR586012	LR585987	LT965021	Castellón, Valencia
*Skoua fertilis*	FMR 15689	LR586001	–	–	LR585988	LT965022	Castellón, Valencia
*Skoua fertilis*	FMR 16032	–	–	–	LR585989	LT965024	Asturias
*Skoua fertilis*	FMR 16320	–	–	–	LR585990	LT965025	Zamora, Castilla y León
*Skoua fertilis*	FMR 16492	–	–	–	LR585991	LT965026	Cáceres, Extremadura
*Skoua fertilis*	FMR 16571	LR586002	–	LR586013	LR585992	LT965027	Badajoz, Extremadura
*Strongyloarthrosporum catenulatum* ^T^	FMR 16121 = CBS 143841	–	–	–	–	LT906534	Toledo, Castilla-La Mancha
*Talaromyces affinitatimellis*	FMR 15674	LT965001	–	–	–	LT968852	Tarragona, Catalonia
*Talaromyces affinitatimellis*	FMR 15675	LT965002	–	–	–	LT968853	Tarragona, Catalonia
*Talaromyces affinitatimellis*	FMR 15677	LT965003	–	–	–	LT968854	Tarragona, Catalonia
*Talaromyces affinitatimellis*	FMR 15684	LT965004	–	–	–	LT968855	Castellón, Valencia
*Talaromyces affinitatimellis*	FMR 15688	LT906553	LT906550	LT906547	LT906538	LT964941	Castellón, Valencia
*Talaromyces affinitatimellis* ^T^	FMR 15690 = CBS 143840	LT906552	LT906549	LT906546	LT906543	LT964939	Castellón, Valencia
*Talaromyces affinitatimellis*	FMR 16029	LT965005	–	–	–	LT968856	Cáceres, Extremadura
*Talaromyces affinitatimellis*	FMR 16033	LT906554	LT906551	LT906548	LT906539	LT964942	Salamanca, Castilla y León
*Talaromyces affinitatimellis*	FMR 16114	LT965006	–	–	–	LT968857	Salamanca, Castilla y León
*Talaromyces affinitatimellis*	FMR 16125	LT965009	–	–	–	LT968860	Zamora, Castilla y León
*Talaromyces affinitatimellis*	FMR 16126	LT965012	–	–	–	LT968861	Zamora, Castilla y León
*Talaromyces affinitatimellis*	FMR 16276	LT965010	–	–	–	LT968862	Zamora, Castilla y León
*Talaromyces affinitatimellis*	FMR 16494	LT965011	–	–	–	LT968863	Zamora, Castilla y León
*Talaromyces affinitatimellis*	FMR 16499	LT965007	–	–	–	LT968858	Cáceres, Extremadura
*Talaromyces affinitatimellis*	FMR 16501	LT965008	–	–	–	LT968859	Cáceres, Extremadura
*Talaromyces basipetosporus* ^T^	FMR 9720 = CBS 143836	LT906563	–	LT906545	LT906542	LT964940	Buenos Aires, Argentina
*Talaromyces brunneosporus* ^T^	FMR 16566 = CBS 144320	LT962483	LT962488	LT962485	LT962487	LT964943	Salamanca, Castilla y León
*Xerochrysium xerophilum*	FMR 15669	–	–	–	LT986724	LT986675	Castellón, Valencia
*Zygosaccharomyces gambellarensis*	FMR 16277	–	–	–	–	LT963549	Salamanca, Castilla y León
*Zygosaccharomyces gambellarensis*	FMR 16569	–	–	–	–	LT963548	Cáceres, Extremadura
*Zygosaccharomyces mellis*	FMR 16280	–	–	–	–	LT963550	Ourense, Galicia
*Zygosaccharomyces mellis*	FMR 16312	–	–	–	–	LT963551	Ourense, Galicia
*Zygosaccharomyces siamensis*	FMR 16034	–	–	–	LT963547	LT984543	Salamanca, Castilla y León

FMR = Faculty of Medicine of Reus culture collection; CBS = Westerdijk Fungal Biodiversity Institute (ex Centraalbureau voor Schimmelcultures). ^T^ = ex type

### Phylogenetic analysis

A preliminary molecular identification of the isolates was carried out with LSU sequences using Basic Local Alignment Search Tool (BLAST; https://blast.ncbi.nlm.nih.gov/Blast.cgi) and only the type sequences or reliable reference strains from GenBank were considered for identification, and a maximum level of identity (MLI) of ≥98% was used for identification at the rank of species and < 98% at the rank of genus. *BenA* for to the genera *Aspergillus*, *Penicillium,* and *Talaromyces*, and ITS for the genera *Monascus*, *Oidiodendron* and *Skoua* were used for identification at the rank of species. An LSU tree was built to determine the phylogenetic relationships of all our isolates. Phylogenetic trees of ITS and a combination of ITS-*BenA*-*CaM*-*rpb*2 were also built to distinguish the members of *Myxotrichaceae* and the genus *Talaromyces,* respectively. *Cunninghamella bertholletiae* (CBS 693.68), *Mucor plumbeus* (DAOM 220743), *Mucor racemosus* (ATCC 42647), and *Rhizopus oryzae* (CBS 112.07 and CBS 130146) were used as outgroup for the LSU tree; *Aphanoascus keratinophilus* (IMI 319010) for the *Myxotrichaceae* taxa tree; and *Trichocoma paradoxa* (CBS 247.57) for the *Talaromyces* tree. The sequence alignments and the maximum-likelihood (ML) and Bayesian-inference (BI) phylogenetic analyses were performed as described previously (Valenzuela-Lopez et al. 2018). The final matrices used for the phylogenetic analysis were deposited in TreeBASE (www.treebase.org; accession number: S23122).

### Growth at different water activities (a_w_)

To test the capacity of growth in different water activities, media containing malt extract (1% w/w), yeast extract (0.25% w/w) and agar-agar (1% w/w) at pH 5.3 were adjusted at six different a_w_ (0.97, 0.95, 0.93, 0.92, 0.88 and 0.82) by adding equal weights of fructose and glucose (corresponding to 22, 30, 40, 44, 48, and 55% w/w of sugars, respectively) (Pitt & Hocking 1977). Water activity was measured in duplicate by a water activity meter (Aqualab, Decagon Devices CX3 02734) with an accuracy of ±0.002 at 25 °C. Triplicate plates were inoculated at their centre with 5 μL of spore suspension of selected fungi, and incubated at 25 °C in darkness, with the exception of FMR 15880, FMR 15883 and FMR 16031, which were at 15 °C (because of their poor growth at 25 °C). The colony diam. was measured after 21 days.

## RESULTS

### Fungal diversity

All honey samples produced fungal colonies on G18 at 15 °C as well as at 25 °C. Table 1 summarizes the fungal strains identified phenotypically and molecularly. With the exception of a few ascomycetous yeasts and of *Mucorales*, most of the fungi were filamentous *Ascomycota*. From the latter, the highest number of strains corresponded to *Skoua* (syn. *Eremascus*) *fertilis*, *Bettsia alvei,* and *Oidiodendron* sp., followed by an unknown arthrosporic fungus*, Eremascus albus* and *Skoua* sp. *Alternaria multiformis*, *Ascosphaera atra*, another unknown arthrospored fungus and *Xerochrysium xerophilum* were isolated only once. Obligate xerophilic species of *Aspergillus* were not found, but the xerotolerant *A. pseudoglaucus*, *A. asperescens* and *A. montevidensis* were isolated. Three species of *Monascus* were identified, i.e. *M. pilosus, M. purpureus,* and *M. ruber*. The isolates of *Penicillium* were classified as *P. camemberti*, *P. citrinum*, *P. corylophilum,* and *P. cravenianum*. Members of *Talaromyces* were classified at the rank of section, i.e. section *Trachyspermi* and section *Purpurei*. We only identified three species of *Mucoromycota*, viz. *Cunninghamella bertholletiae*, *Mucor plumbeus,* and *Rhizopus oryzae*. Regardless of their geographical origin, type of honey (nectar or honeydew) and if honey was or not thermally treated, *S. fertilis* and *B. alvei* were present in all honey samples.

### Molecular phylogeny

**Fig. 2:**
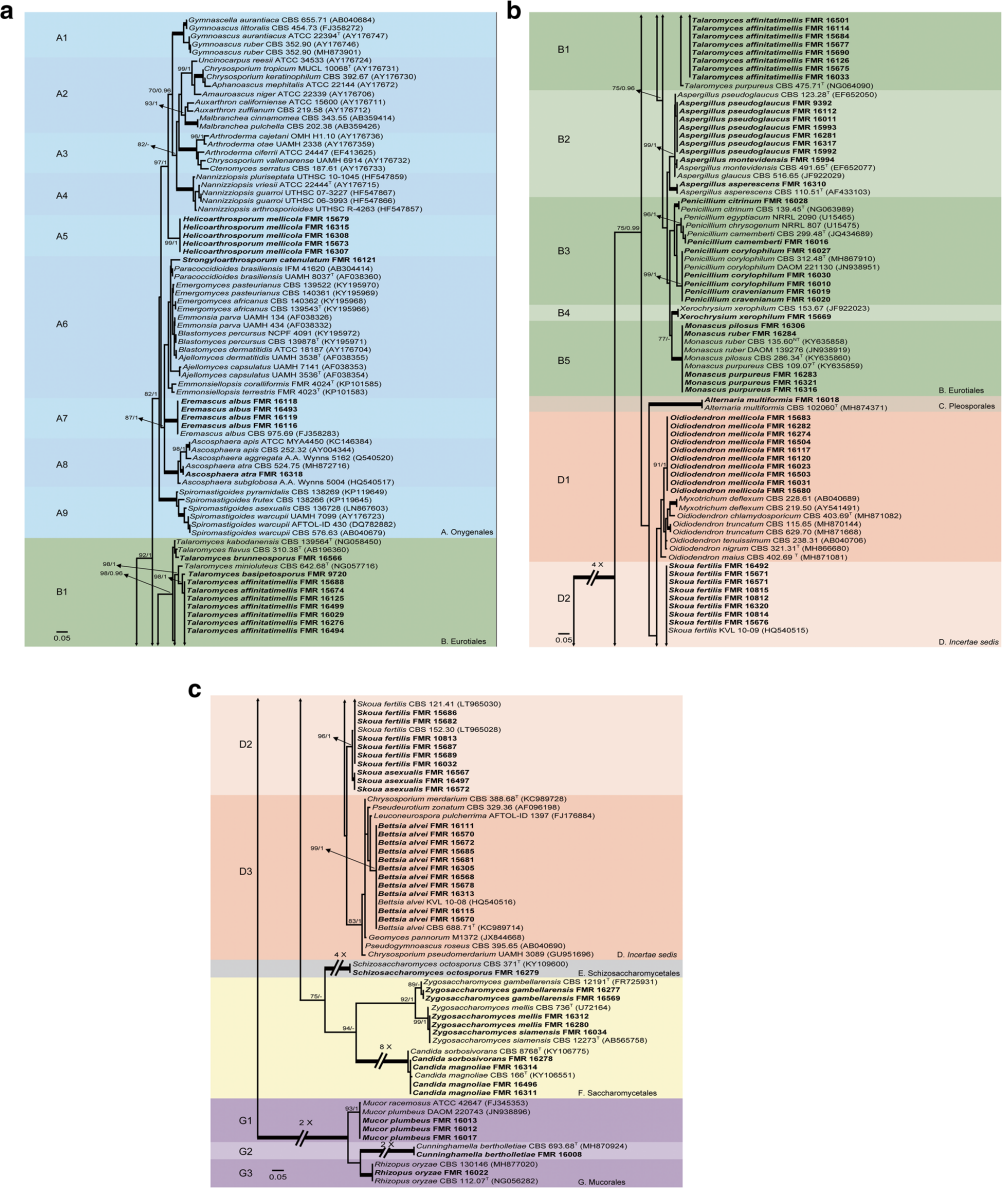
**a**-**c** ML phylogenetic tree based on the analysis of LSU nucleotide sequences for all fungi isolated from honey. Members of Mucoromycota were chosen as out-group. Support in nodes is indicated above thick branches and is represented by posterior probabilities (BI analysis) of 0.95 and higher and/or bootstrap values (ML analysis) of 70% and higher. Fully supported branched (100% BS /1 PP) are indicated in bold. ^T^ = ex type. Alignment length 606 bp. The sequences generated by us are in Table 1

**Fig. 3:**
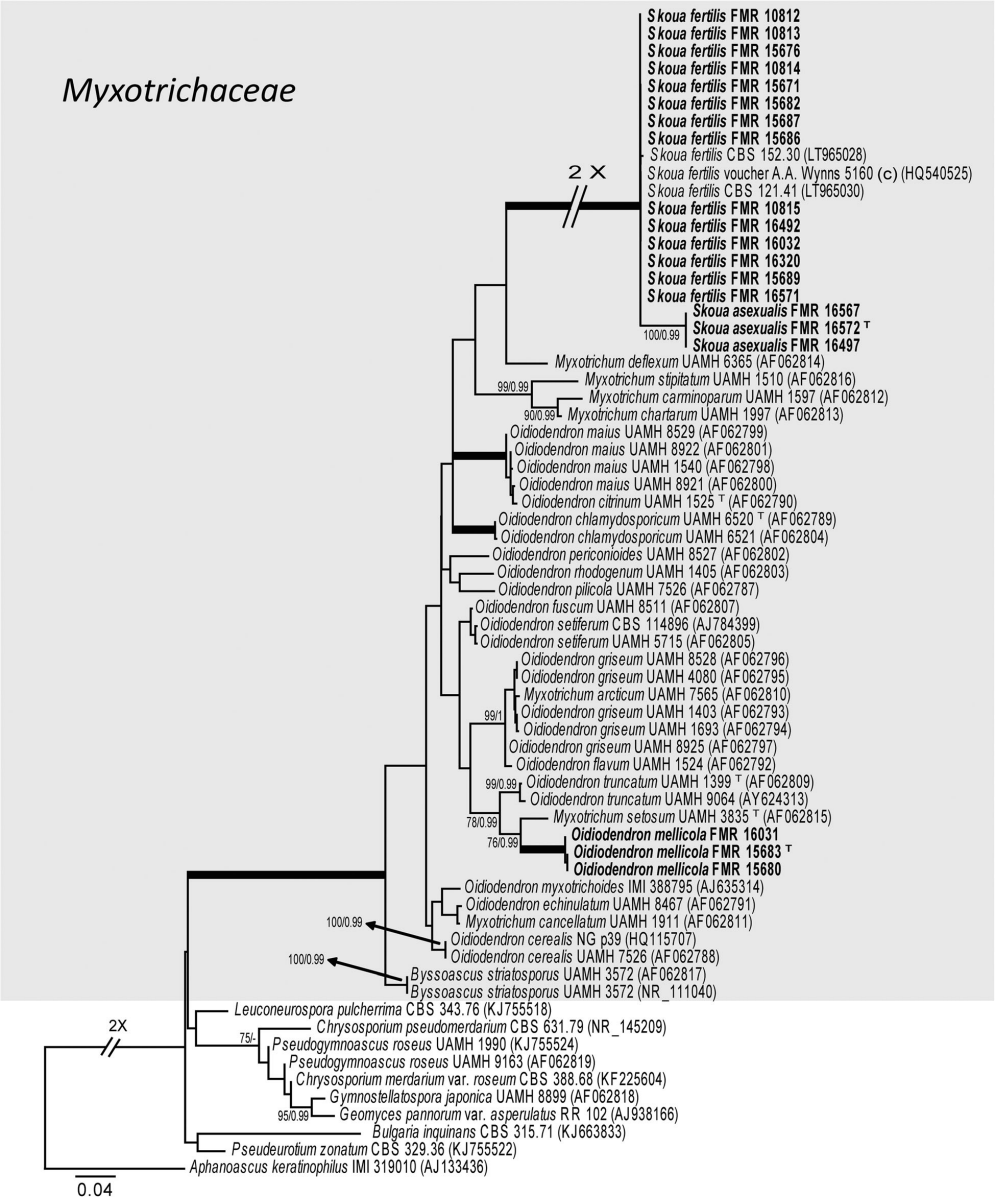
ML phylogenetic tree based on the analysis of ITS nucleotide sequences of representative taxa of the families *Myxotrichaceae* (in grey balckground) and *Pseudeurotiaceae*. *Aphanoascus keratinophilus* IMI 319010 was chosen as out-group. Support in nodes is indicated above thick branches and is represented by posterior probabilities (BI analysis) of 0.95 and higher and/or bootstrap values (ML analysis) of 70% and higher. Fully supported branched (100% BS /1 PP) are indicated in bold. ^T^ = ex type. Alignment length 544 bp

**Fig. 4:**
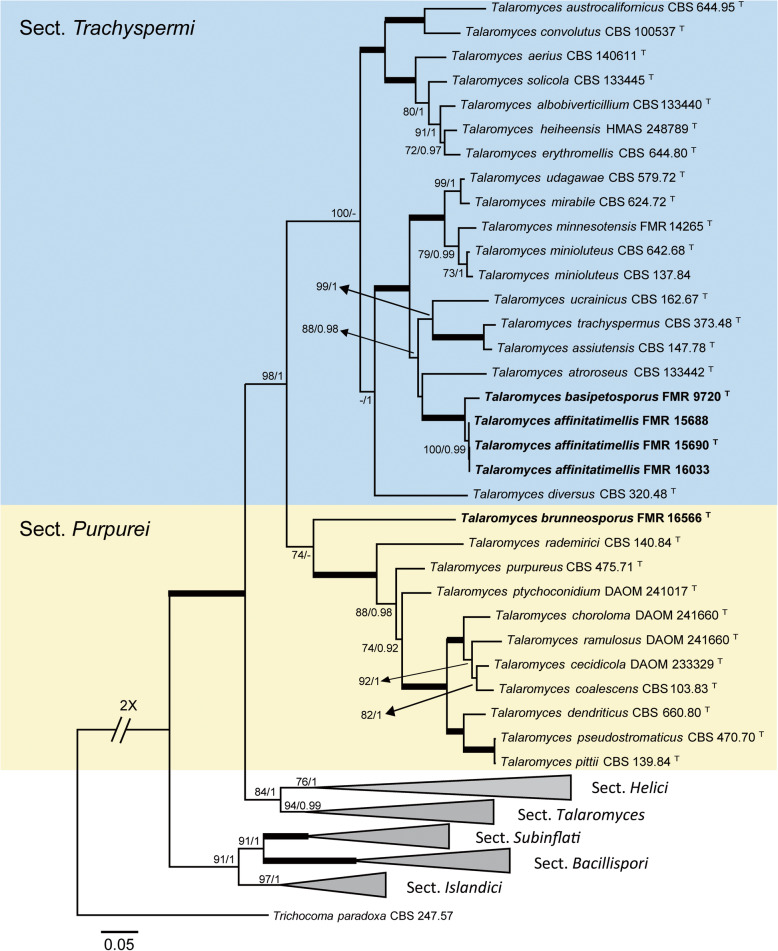
ML phylogenetic tree built using the ITS, *BenA*, *CaM* and *rpb*2 concatenated dataset for species of the genus *Talaromyces*. Species of the section *Trachyspermi* are indicated in a blue background and those of the section *Purpurei* in yellow. *Trichocoma paradoxa* CBS 247.57 was chosen as out-group. Support in nodes is indicated above thick branches and is represented by posterior probabilities (BI analysis) of 0.95 and higher and/or bootstrap values (ML analysis) of 70% and higher. Fully supported branched (100% BS /1 PP) are indicated in bold. ^T^ = ex-type strain. Alignment length 2265 bp

Our first phylogenetic study included 206 LSU sequences with a total of 606 characters, including gaps, 352 of them being parsimony informative. The ML analysis was congruent with that obtained in the BI analysis, both displaying trees with similar topologies. The isolates were distributed across two main clades (Fig. 2a-c), the first (100% BS / 1 PP) corresponding to the *Ascomycota* and including 99 isolates, and the second (100% BS / 1 PP) involving the rest of the isolates and pertaining to the *Mucoromycota*. The first main clade was divided into six subclades: A (82% BS / 1 PP), which represents *Onygenales*; B (75% BS / 0.96 PP), *Eurotiales*; C (100% BS / 1 PP); *Pleosporales,* D (unssuported) as *incertae sedis*; E (100% BS / 1 PP), *Schizosaccharomycetales*, and F (94% BS / - PP), *Saccharomycetales*. Subclade A contains seven well-supported groups, six of which represent the known families of *Onygenales*, i.e. *Gymnoascaceae* (A1), *Arthrodermataceae* (A3), *Nannizziopsiaceae* (A4), *Eremascaceae* (A7), *Ascosphaeriaceae* (A8), and *Spiromastigaceae* (A9), and a seventh group (A5) composed of five of our strains probably representing a new family. The groups representing *Ajellomycetaceae* (A6) and *Onygenaceae* (A2) were unsupported. Strains in subclade A were distributed as follows: the five mentioned above into A5, FMR 16121 into a separate branch of the *Ajellomycetaceae* (A6), four strains conspecific with *Eremascus albus* (A7), and one (FMR 16318) identified as *Ascosphaera atra* (A8). Thirty-nine strains were placed in *Eurotiales* (Subclade B). One (FMR 16566) was placed together with *Talaromyces flavus* and *T. kabodanensis* in an unsupported branch, and 16 strains near to *T. minioluteus* into a well-supported sister clade (B1). Into B2 (unsupported), which includes species of *Aspergillus*, eight of the strains were placed in a branch (99% BS / 1 PP) together with *A. glaucus, A. montevidensis* and *A. pseudoglaucus* (sect. *Aspergillus*). For the final identification of these eight strains, we used *Ben*A sequence comparison, which were found to be *A. montevidensis* (one strain) and *A. pseudoglaucus* (seven strains). FMR 16310 was placed in a branch together with the ex-type sequence of *A. asperescens* (sect. *Nidulantes*). Seven strains grouped into the sister clade B3 (unsupported), representing five species of *Penicillium*. FMR 15669 was identified as *Xerochrysium xerophilum* (B4), and five strains were initially identified as *Monascus* spp. Based on the comparison of ITS sequences, these five strains were finally identified as *M. pilosus* (one strain), *M. purpureus* (three strains), and *M. ruber* (one strain). Strain FMR 16018 was located together with *Alternaria multiformis* (Subclade C, *Pleosporales*). Subclade D (unsupported) was divided into three groups: D1, representing the *Myxotrichaceae*; D2, the genus *Skoua*; and D3, the *Pseudeurotiaceae*. This group had 38 strains, 10 among the genera *Oidiodendron* and *Myxotrichum* (D1), 17 together with *Skoua fertilis* (D2), and 11 within *Bettsia alvei* (D3). Subclade E (*Schizosaccharomycetales*), grouped FMR 16279 together with the ex-type sequence of *Schizosaccharomyces octosporus*. Subclade F (*Saccharomycetales*), had nine strains belonging to *Zygosaccharomyces* spp. (five strains) and *Candida* spp. (four strains). Clade G had 5 strains, *Mucorales*, divided into three groups that comprised *Mucor* spp. (three strains)*, Cunninghamella bertholletiae* (FMR 16008) and *Rhizopus oryzae* (FMR 16022), respectively. Figures 3, 4 show the trees resulting from the phylogenetic analyses of *Myxotrichaceae* and *Talaromyces*, respectively. The phylogenetic tree based on the analysis of the ITS (Fig. 3), included 67 sequences belonging to *Myxotrichaceae* and *Pseudeurotiaceae*, whose alignments encompassed a total of 547 characters, including gaps, 204 of which were parsimony informative. The ML and BI analyses showed a similar tree topology. It comprised a main clade of *Myxotrichaceae*, where 20 strains were located, 17 of *Skoua* (14 identified as *S. fertilis*), and the remaining three in a separate branch that might represent a new species of the genus. Finally, three strains phylogenetically distant from the others appeared in a separate branch close to *Myxotrichum setosum* and *Oidiodendron truncatum*. The tree based on four concatenated *loci* (*BenA*, *CaM*, *rpb2* and ITS; Table 2; Fig. 4) was built to resolve the phylogenetic relationships of the *Talaromyces* strains. The dataset contained 123 sequences with a total of 2265 characters, including gaps, (520 of them for ITS, 377 for *BenA*, 516 for *CaM* and 852 for *rpb*2), of which 1069 were parsimony informative (195 for ITS, 217 for *BenA*, 308 for *CaM* and 349 for *rpb*2). The sequence datasets did not show conflict in the tree topologies for the 70% reciprocal bootstrap trees, which allowed the multi-locus analysis. The ML analysis showed similar tree topology and was congruent with the Bayesian analysis. In this tree (Fig. 4), the five *Talaromyces* strains we obtained were located in two different clades: one corresponding to the section *Trachyspermi* (100% BS / - PP), with four strains phylogenetically distant from *T. atroroseus*, one of them (FMR 9720) in a separate branch; and the second corresponding to the section *Purpurei* (74% BS / - PP), where the fifth strain (FMR 16566) was located in a distant branch.

**Table 2:** Talaromyces spp. nucleotide sequences employed to build a phylogram to locate phylogenetically our strains from honey

Species name	Section	Strain no.	GenBank accession #			
			*BenA*	*CaM*	*rpb2*	*ITS*
*Talaromyces aculeatus*	*Talaromyces*	CBS 289.48 = IMI 040588 = NRRL 2129	KF741929	KF741975	KM023271	KF741995
*Talaromyces adpressus*	*Talaromyces*	CBS 140620 = CGMCC3.18211 = DTO 317-G4	KU866844	KU866741	KU867001	KU866657
*Talaromyces alveolaris*	*Talaromyces*	UTHSC DI16–146	LT559085	LT795594	LT795595	LT558968
*Talaromyces amazonensis*	*Talaromyces*	CBS 140373 = IBT 23215 = DTO 093-F9	KX011490	KX011502	–	KX011509
*Talaromyces amestolkiae*	*Talaromyces*	CBS 132696 = DTO 179-F5	JX315623	KF741937	JX315698	JX315660
*Talaromyces angelicae*	*Talaromyces*	KACC 46611	KF183640	KJ885259	–	KF183638
*Talaromyces apiculatus*	*Talaromyces*	CBS 312.59 = FRR 635 = IMI 068239	KF741916	KF741950	KM023287	JN899375
*Talaromyces aurantiacus*	*Talaromyces*	CBS 314.59 = IMI 099722 = NRRL 3398	KF741917	KF741951	–	JN899380
*Talaromyces beijingensis*	*Talaromyces*	CBS 140617 = CGMCC3.18200 = DTO 317-D8	KU866837	KU866733	KU866993	KU866649
*Talaromyces calidicanius*	*Talaromyces*	CBS 112002	HQ156944	KF741934	KM023311	JN899319
*Talaromyces cnidii*	*Talaromyces*	KACC 46617	KF183641	KJ885266	KM023299	KF183639
*Talaromyces derxii*	*Talaromyces*	CBS 412.89	JX494305	KF741959	KM023282	JN899327
*Talaromyces duclauxii*	*Talaromyces*	CBS 322.48 = IMI 040044 = MUCL 28672 = NRRL 1030	JX091384	KF741955	JN121491	JN899342
*Talaromyces euchlorocarpius*	*Talaromyces*	PF 1203 = DTO 176-I3 = DTO 176-I4	KJ865733	KJ885271	KM023303	AB176617
*Talaromyces flavovirens*	*Talaromyces*	CBS 102801 = IBT 27044	JX091376	KF741933	–	JN899392
*Talaromyces flavus*	*Talaromyces*	CBS 310.38 = IMI 197477 = NRRL 2098	JX494302	KF741949	JF417426	JN899360
*Talaromyces francoae*	*Talaromyces*	CBS 113134 = IBT 23221 = DTO 056-D9	KX011489	KX011501	–	KX011510
*Talaromyces funiculosus*	*Talaromyces*	CBS 272.86 = IMI 193019	JX091383	KF741945	KM023293	JN899377
*Talaromyces fusiformis*	*Talaromyces*	CBS 140637 = CGMCC3.18210 = DTO 317-F4	KU866843	KU866740	KU867000	KU866656
*Talaromyces galapagensis*	*Talaromyces*	CBS 751.74 = IFO 31796	JX091388	KF741966	–	JN899358
*Talaromyces indigoticus*	*Talaromyces*	CBS 100534 = IBT 17590	JX494308	KF741931	–	JN899331
*Talaromyces intermedius*	*Talaromyces*	CBS 152.65 = BDUN 267 = IFO 31752 = IMI 100874	JX091387	KJ885290	–	JN899332
*Talaromyces kabodanensis*	*Talaromyces*	DI16–149	LT559088	LT795598	LT795599	LT558971
*Talaromyces liani*	*Talaromyces*	CBS 225.66 = IMI 098480 = NRRL 3380 = VKM F-301	JX091380	KJ885257	–	JN899395
*Talaromyces macrosporus*	*Talaromyces*	CBS 317.63 = FRR 404 = IMI 197478	JX091382	KF741952	KM023292	JN899333
*Talaromyces mangshanicus*	*Talaromyces*	CGMCC 3.18013	KX447530	KX447528	KX447527	KX447531
*Talaromyces marneffei*	*Talaromyces*	CBS 388.87	JX091389	KF741958	KM023283	JN899344
*Talaromyces muroii*	*Talaromyces*	CBS 756.96 = PF 1153	KJ865727	KJ885274	–	JN899351
*Talaromyces neofusisporus*	*Talaromyces*	AS3.15415 = CBS 139516	KP765381	KP765383	–	KP765385
*Talaromyces oumae-annae*	*Talaromyces*	CBS 138208 = DTO 269-E8	KJ775213	KJ775425	–	KJ775720
*Talaromyces panamensis*	*Talaromyces*	CBS 128.89 = IMI 297546	HQ156948	KF741936	KM023284	JN899362
*Talaromyces paucisporus*	*Talaromyces*	PF 1150 = IFM 53616	–	–	–	AB176603
*Talaromyces pinophilus*	*Talaromyces*	CBS 631.66 = CECT 2809 = DSM 1944 = IAM 7013 = IMI 114933	JX091381	KF741964	KM023291	JN899382
*Talaromyces primulinus*	*Talaromyces*	CBS 321.48 = CBS 439.88 = FRR 1074 = IMI 040031 = MUCL 31321 = NRRL 1074	JX494305	KF741954	KM023294	JN899317
*Talaromyces purgamentorum*	*Talaromyces*	CBS 113145 = IBT 23220 = DTO 056-E1	KX011487	KX011500	–	KX011504
*Talaromyces purpurogenus*	*Talaromyces*	CBS 286.36 = IMI 091926	JX315639	KF741947	JX315709	JN899372
*Talaromyces qii*	*Talaromyces*	AS3.15414 = CBS 139515	KP765380	KP765382	–	KP765384
*Talaromyces rapidus*	*Talaromyces*	UTHSC DI16–148 = CBS 142382 T	LT559087	LT795600	LT795601	LT558970
*Talaromyces ruber*	*Talaromyces*	CBS 132704 = DTO 193-H6 = IBT 10703 = CBS 113137	JX315629	KF741938	JX315700	JX315662
*Talaromyces rubicundus*	*Talaromyces*	CBS 342.59 = IMI 099723 = NRRL 3400	JX494309	KF741956	KM023296	JN899384
*Talaromyces sayulitensis*	*Talaromyces*	CBS 138204 = DTO 245-H1	KJ775206	KJ775422	–	KJ775713
*Talaromyces siamensis*	*Talaromyces*	CBS 475.88 = IMI 323204	JX091379	KF741960	KM023279	JN899385
*Talaromyces stipitatus*	*Talaromyces*	CBS 375.48 = NRRL 1006 = IMI 39805	KM111288	KF741957	KM022380	JN899348
*Talaromyces stollii*	*Talaromyces*	CBS 408.93	–	JX315646	JX315712	JX315674
*Talaromyces thailandensis*	*Talaromyces*	CBS 133147 = KUFC 3399	JX494294	KF741940	KM023307	JX898041
*Talaromyces verruculosus*	*Talaromyces*	CBS 388.48 = DSM 2263 = IMI 040039 = NRRL 1050	KF741928	KF741944	KM023306	KF741994
*Talaromyces viridis*	*Talaromyces*	CBS 114.72 = ATCC 22467 = NRRL 5575	JX494310	KF741935	JN121430	AF285782
*Talaromyces viridulus*	*Talaromyces*	CBS 252.87 = FRR 1863 = IMI 288716	JX091385	KF741943	JF417422	JN899314
*Talaromyces aerugineus*	*Helici*	CBS 350.66 = BDUN 276 = IMI 105412	KJ865736	KJ885285	JN121502	AY753346
*Talaromyces bohemicus*	*Helici*	CBS 545.86 = CCF 2330 = IAM 14789	KJ865719	KJ885286	JN121532	JN899400
*Talaromyces boninensis*	*Helici*	CBS 650.95 = IBT 17516	KJ865721	KJ885263	KM023276	JN899356
*Talaromyces cinnabarinus*	*Helici*	CBS 267.72 = NHL 2673	AY753377	KJ885256	JN121477	JN899376
*Talaromyces diversiformis*	*Helici*	CBS 141931 = CGMCC3.18204 = DTO 317-E3	KX961216	KX961259	KX961274	KX961215
*Talaromyces georgiensis*	*Helici*	UTHSC DI16–145 = CBS 142380	LT559084	–	LT795606	LT558967
*Talaromyces helicus*	*Helici*	CBS 335.48 = DSM 3705 = IMI 040593 = NRRL 2106	KJ865725	KJ885289	KM023273	JN899359
*Talaromyces reverso-olivaceus*	*Helici*	CBS 140672 = CGMCC3.18195 = DTO 317-C3	KU866834	KU866730	KU866990	KU866646
*Talaromyces ryukyuensis*	*Helici*	NHL 2917 = DTO 176-I6	–	–	–	AB176628
*Talaromyces varians*	*Helici*	CBS 386.48 = IMI 040586 = NRRL 2096	KJ865731	KJ885284	KM023274	JN899368
*Talaromyces cecidicola*	*Purpurei*	CBS 101419 = DAOM 233329	FJ753295	KJ885287	KM023309	AY787844
*Talaromyces chlorolomus*	*Purpurei*	DAOM 241016 = CV 2802	GU385736	KJ885265	KM023304	FJ160273
*Talaromyces coalescens*	*Purpurei*	CBS 103.83	JX091390	KJ885267	KM023277	JN899366
*Talaromyces dendriticus*	*Purpurei*	CBS 660.80 = IMI 216897	JX091391	KF741965	KM023286	JN899339
*Talaromyces pittii*	*Purpurei*	CBS 139.84 = IMI 327871	KJ865728	KJ885275	KM023297	JN899325
*Talaromyces pseudostromaticus*	*Purpurei*	CBS 470.70 = FRR 2039	HQ156950	KJ885277	KM023298	JN899371
*Talaromyces ptychoconidium*	*Purpurei*	DAOM 241017 = CV 2808 = DTO 180-E7	GU385733	JX140701	KM023278	FJ160266
*Talaromyces purpureus*	*Purpurei*	CBS 475.71 = FRR 1731 = IMI 181546	GU385739	KJ885292	JN121522	JN899328
*Talaromyces rademirici*	*Purpurei*	CBS 140.84 = CECT 2771 = IMI 282406	KJ865734	–	KM023302	JN899386
*Talaromyces ramulosus*	*Purpurei*	DAOM 241660 = CV 2837 = DTO 184-B8	FJ753290	JX140711	KM023281	EU795706
*Talaromyces aerius*	*Trachyspermi*	CBS 140611 = CGMCC3.18197 = DTO 317-C7	KU866835	KU866731	KU866991	KU866647
*Talaromyces albobiverticillius*	*Trachyspermi*	CBS 133440 T = DTO 166-E5 = YMJ 1292	KF114778	KJ885258	KM023310	HQ605705
*Talaromyces assiutesis*	*Trachyspermi*	CBS 147.78 T	KJ865720	KJ885260	KM023305	N899323
*Talaromyces atroroseus*	*Trachyspermi*	CBS 133442 T = IBT 32470 = DTO 178-A4	KF114789	KJ775418	KM023288	KF114747
*Talaromyces austrocalifornicus*	*Trachyspermi*	CBS 644.95 T = IBT 17522	KJ865732	KJ885261	–	JN899357
*Talaromyces convolutus*	*Trachyspermi*	CBS 100537 T = IBT 14989	KF114773	–	JN121414	JN899330
*Talaromyces diversus*	*Trachyspermi*	CBS 320.48 T = DSM 2212 = IMI 040579 = NRRL 2121	KJ865723	KJ885268	KM023285	KJ865740
*Talaromyces erythromellis*	*Trachyspermi*	CBS 644.80 T = FRR 1868 = IMI 216899	HQ156945	KJ885270	KM023290	JN899383
*Talaromyces heiheensis*	*Trachyspermi*	CGMCC 3.18012	KX447525	KX447532	KX447529	KX447526
*Talaromyces minioluteus*	*Trachyspermi*	CBS 137.84	KF114798	–	–	NR138301
*Talaromyces minioluteus*	*Trachyspermi*	CBS 642.68 = IMI 089377 = MUCL 28666	KF114799	KJ885273	JF417443	JN899346
*Talaromyces minnesotensis*	*Trachyspermi*	FMR 14265 T = CBS 142381	LT559083	LT795604	LT795605	LT558966
*Talaromyces mirabile*	*Trachyspermi*	CBS 624.72	KF114797	–	–	NR138300
*Talaromyces solicola*	*Trachyspermi*	DAOM 241015 T = CV 2800 = DTO 180-D4	GU385731	KJ885279	KM023295	FJ160264
*Talaromyces trachyspermus*	*Trachyspermi*	CBS 373.48 T = IMI 040043	KF114803	KJ885281	JF417432	JN899354
*Talaromyces ucrainicus*	*Trachyspermi*	CBS 162.67 T = FRR 3462 = NHL 6086	KF114771	KJ885282	KM023289	JN899394
*Talaromyces udagawae*	*Trachyspermi*	CBS 579.72 T = FRR 1727 = IMI 197482	KF114796	–	–	JN899350
*Talaromyces bacillisporus*	*Bacillispori*	CBS 296.48 = IMI 040045 = NRRL 1025	AY753368	KJ885262	JF417425	KM066182
*Talaromyces columbiensis*	*Bacillispori*	CBS 113151 = IBT 23206 = DTO 058-F3	KX011488	KX011499	–	KX011503
*Talaromyces emodensis*	*Bacillispori*	CBS 100536 = IBT 14990	KJ865724	KJ885269	JN121552	JN899337
*Talaromyces hachijoensis*	*Bacillispori*	PF 1174 = IFM 53624	–	–	–	AB176620
*Talaromyces mimosinus*	*Bacillispori*	CBS 659.80 = FRR 1875 = IMI 223991	KJ865726	KJ885272	–	JN899338
*Talaromyces proteolyticus*	*Bacillispori*	CBS303.67 = NRRL 3378	KJ865729	KJ885276	KM023301	JN899387
*Talaromyces unicus*	*Bacillispori*	CBS 100535 = CCRC 32703 = IBT 18385	KJ865735	KJ885283	–	JN899336
*Talaromyces palmae*	*Subinflati*	CBS 442.88 = IMI 343640	HQ156947	KJ885291	KM023300	JN899396
*Talaromyces subinflatus*	*Subinflati*	CBS 652.95 = IBT 17520	KJ865737	KJ885280	KM023308	JN899397
*Talaromyces acaricola*	*Islandici*	CBS 137386 = DTO 183-B3 = DAOM 241025 = IBT 32387	JX091610	JX140729	KF984956	JX091476
*Talaromyces allahabadensis*	*Islandici*	CBS 304.63	KF984614	KF984768	KF985006	KF984873
*Talaromyces atricola*	*Islandici*	CBS 255.31 = NRRL 1052 = FRR 1052 = Thom 4640.439	KF984566	KF984719	KF984948	KF984859
*Talaromyces brunneus*	*Islandici*	CBS 227.60 = FRR 646 = IFO 6438 = IHEM 3907 = IMI 078259 = MUCL 31318	KJ865722	KJ885264	KM023272	JN899365
*Talaromyces cerinus*	*Islandici*	CBS 140622 = CGMCC3.18212 = DTO 318-A2	KU866845	KU866742	KU867002	KU866658
*Talaromyces chlamydosporus*	*Islandici*	CBS 140635 = CGMCC3.18199 = DTO 317-D5	KU866836	KU866732	KU866992	KU866648
*Talaromyces columbinus*	*Islandici*	NRRL 58811	KF196843	KJ885288	KM023270	KJ865739
*Talaromyces crassus*	*Islandici*	CBS 137381 = DTO 181-C5 = DAOM 241027 = IBT 32814	JX091608	JX140727	KF984914	JX091472
*Talaromyces infraolivaceus*	*Islandici*	CBS 137385 = DTO 182-I2 = DAOM 241024 = IBT 32487	JX091615	JX140734	KF984949	JX091481
*Talaromyces islandicus*	*Islandici*	CBS 338.48 = IMI 040042 = MUCL 31324 = NRRL 1036	KF984655	KF984780	KF985018	KF984885
*Talaromyces loliensis*	*Islandici*	CBS 643.80 = FRR 1798 = IMI 216901 = MUCL 31325	KF984658	KF984783	KF985021	KF984888
*Talaromyces neorugulosus*	*Islandici*	CBS 140623 = CGMCC3.18215 = DTO 318-A8	KU866846	KU866743	KU867003	KU866659
*Talaromyces piceus*	*Islandici*	CBS 361.48 = IMI 040038 = NRRL 1051	KF984668	KF984680	KF984899	KF984792
*Talaromyces radicus*	*Islandici*	CBS 100489 = FRR 4718	KF984599	KF984773	KF985013	KF984878
*Talaromyces rotundus*	*Islandici*	CBS 369.48 = IMI 040589 = NRRL 2107	KJ865730	KJ885278	KM023275	JN899353
*Talaromyces rugulosus*	*Islandici*	CBS 371.48 = IMI 040041 = MUCL 31201 = NRRL 1045	KF984575	KF984702	KF984925	KF984834
*Talaromyces scorteus*	*Islandici*	CBS 340.34 = NRRL 1129 = FRR 1129	KF984565	KF984684	KF984916	KF984892
*Talaromyces subaurantiacus*	*Islandici*	CBS 137383 = DTO 181-I2 = DAOM 241020 = IBT 32838	JX091609	JX140728	KF984960	LT558965
*Talaromyces tardifaciens*	*Islandici*	CBS 250.94	KC202954	KF984682	KF984908	JN899361
*Talaromyces tratensis*	*Islandici*	CBS 133146 = KUFC 3383	KF984559	KF984690	KF984911	KF984891
*Talaromyces wortmannii*	*Islandici*	CBS 391.48 = IMI 040047 = NRRL 1017	KF984648	KF984756	KF984977	KF984829
*Talaromyces yelensis*	*Islandici*	DTO 268E5	KJ775210	–	–	KJ775717
*Trichocoma paradoxa*	–	CBS 247.57	JF417468	JF417505	JF417421	JF417485

## TAXONOMY

### Subclade A: *Onygenales*

Based on the above phylogenetic analyses, we suggest the following novel taxonomic arrangements: *Helicoarthrosporaceae* fam. nov. (Fig. 2; sister clade A5), phylogenetically close to the family *Gymnoascaceae,* with *Helicoarthrosporum* gen. nov. as type genus and *H. mellicola* sp. nov. as the type species; based on the strain FMR 16121, we introduce *Strongyloarthrosporum* gen. nov. with *S. catenulatum* sp. nov. as its type species. These new taxa are described and illustrated below.

Helicoarthrosporaceae Stchigel, Rodr.-Andr. & Cano, fam. nov. MycoBank MB 832226.

*Diagnosis:* Differing from other families of Onygenales by the production of long, sinuous to helical chains of arthroconidia (which are shorter, right, curved or contorted in other taxa).

*Type genus: Helicoarthrosporum* Stchigel et al. 2019.

*Description*: *Hyphae* hyaline, septate. *Asexual morph* reduced to sinuous, helical or zig-zag lateral branches, terminal part becoming fertile, disarticulating into conidia. *Conidia* hyaline, prismatic to cuboid, holo- and enteroarthric conidia. *Sexual morph* not observed.

Helicoarthrosporum Stchigel, Cano & Rodr.-Andr., gen. nov. MycoBank MB 823584.

*Etymology.* From Greek *έλικα*-, helix, −*άρθρωση-*, joint, and -*σπορά*, spore, referring to the morphology of the conidiophores.

*Diagnosis*: Distinguished from other phylogenetically related genera by its long, sinuous to helical chains of prismatic to cuboid arthroconidia, and by its extreme xerotolerance.

*Type species: Helicoarthrosporum mellicola* Stchigel et al. 2019.

*Description: Mycelium* composed by hyaline, septate hyphae. *Conidiophores* consisting in fertile lateral branches and terminal part of the hyphae, sinuous, helical or zig-zag, disarticulating in hyaline, mostly prismatic to cuboid, holo- and enteroarthric conidia.

**Fig. 5:**
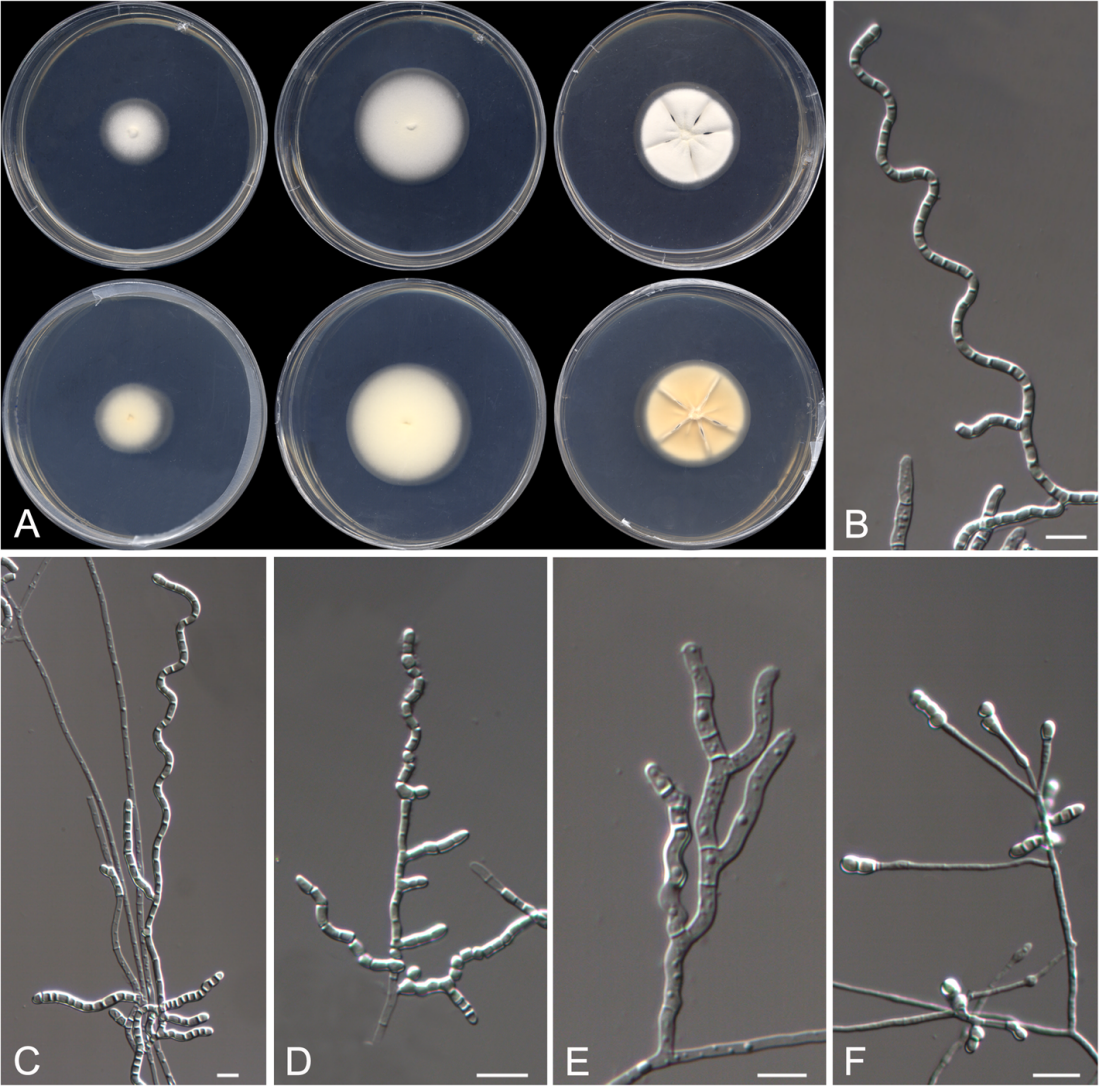
*Helicoarthrosporum mellicola* CBS 143838 ^T^. **a** Colonies on G18 at 15 °C and at 25 °C, and on PDA at 25 °C (from left to right), surface and reverse (from top to bottom). **b**–**e** Conidiophores. **d** Chlamydospores on OA. Scale bar = 10 μm

Helicoarthrosporum mellicola Stchigel, Cano & Rodr.-Andr., sp. nov. Fig. 5. MycoBank MB 823585.

*Etymology:* From Latin *mellis*-, honey, and -*cola*, to reside, referring to the habitat of the fungus.

*Diagnosis: Helicoarthrosporum mellicola* morphologically resembles *Scytalidium cuboideum* (syn. *Arthrographis cuboidea*), *S. ganodermophthorum,* and *S. sphaerosporum* in producing long chains of cuboid arthroconidia (Kang et al. 2010). *Helicoarthrosporum mellicola* grows slowly on PDA and shows a high xerotolerance, whereas *Scytalidium* spp. grow fast on PDA and do not show a xerotrophic habit; also, *S. ganodermophthorum* and S. *sphaerosporum* produce both asexual and sexual morphs, while *H. mellicola* only displays an asexual one.

*Type:* Spain: *Valencia community*: Castellón province, from decanted and filtered honey, 10 May 2014, *A. Gómez Pajuelo* (CBS H-23368 – holotype; CBS 143838 = FMR 15679 – ex-type cultures; LSU sequence GenBank LT906535).

*Description: Colonies* on G18 reaching 38–41 mm diam after 3 wk. at 25 °C, flattened, velvety, yellowish white (4A2) at the centre, margins regular, sporulation sparse; exudate absent; reverse pale yellow (4A3), diffusible pigment absent. *Mycelium* composed of hyaline to subhyaline, septate, smooth- and thin-walled hyphae, 1.5–4 μm wide; racquet hyphae present. *Conidiophores* reduced (mostly) to fertile side branches and to the terminal part of a vegetative hyphae, sinuous to helical or in zig-zag, mostly simple, sometimes branched, 15–180 μm long, hyaline, disarticulating in conidia. *Conidia* mostly 1-celled, sometimes up to 4-celled, mostly holoarthric, occasionally enteroarthric, in chains of up to 30, mostly barrel-shaped, prismatic or cuboid, sometimes triangular and “Y”-shaped, smooth-walled, thicker than the hyphae, thickener at the ends, 2–8 × 2–5 μm, hyaline, disarticulating by schizolysis or rhexolysis from the conidiogenous hyphae. *Chlamydospores* produced on OA, terminally on or intercalary in the fertile hyphae hyaline, one to multicellular, smooth- and thick-walled, globose, ovoid, pyriform, clavate or irregularly-shaped, truncate at the base or at both ends, to 10 μm long and 3–5 μm wide.

*Colonies* on G18 reaching 22–27 mm diam after 3 wk. at 15 °C, flat, velvety, yellowish-white (4A2), margins regular, sporulation sparse, exudate absent; reverse pale yellow (4A3), diffusible pigment absent; no growth on G18 over 35 °C; on PDA reaching 31–35 mm diam after 3 wk. at 25 °C, slightly elevated, velvety, slightly sulcate, yellowish (3A2) at the centre and white (3A1) at the edge, exudate absent; reverse reddish yellow (4A6) at the centre and pale orange (5A3) at the edge, diffusible pigment absent; on OA at 25 °C after 3 wk. very small, 7–8 mm diam, velvety, white (4A1), sporulation sparse, exudate absent; reverse pale orange (5A3), diffusible pigment absent.

Minimum, optimal and maximum temperature of growth on G18 are 15 °C, 25 °C, and 30 °C, respectively; no hemolysis observed on blood agar at 25 °C, and on BCP-MS-G casein hydrolyzed without pH changes. Lipase negative, urease positive. Inhibited by cycloheximide and 20% NaCl, but tolerant to 3% and to 10% NaCl on Sabouraud dextrose agar.

*Other specimens examined:* Spain: *Valencia community*: Castellón province, from decanted and filtered honey, 10 May 2014, *A. Gómez Pajuelo* (FMR 15673). *Castilla y León community*: León province, from decanted, filtered and thermally treated honey, 20 May 2014, *A. Terrab* (FMR 16307). *Castilla y León community*: Zamora province, from decanted and filtered honey, 5 Oct. 2014, *A. Gómez Pajuelo* (FMR 16308). *Extremadura community*: Cáceres province, from decanted, filtered and thermally treated honey, 16 May 2014, *A. Terrab* (FMR 16315).

Strongyloarthrosporum Rodr.-Andr., Cano & Stchigel, gen. nov*.* MycoBank MB 823587.

*Etymology:* From Greek *στρογγυλός-,* globose, −*άρθρωση-*, joint, and -*σπορά*, spore, referring to the morphology of the conidia.

*Diagnosis*: Distinguished from other genera of Onygenales by the production of thick-walled globose arthroconidia, and because this fungus is an obligate xerophile.

*Type species: Strongyloarthrosporum catenulatum* Rodr.-Andr. et al. 2019.

*Description*: *Mycelium* of hyaline, septate hyphae. *Conidiophores* fertile lateral branches and part of the vegetative hyphae, disarticulating. *Conidia* enteroarthic, hyaline, mostly globose*.*

**Fig. 6:**
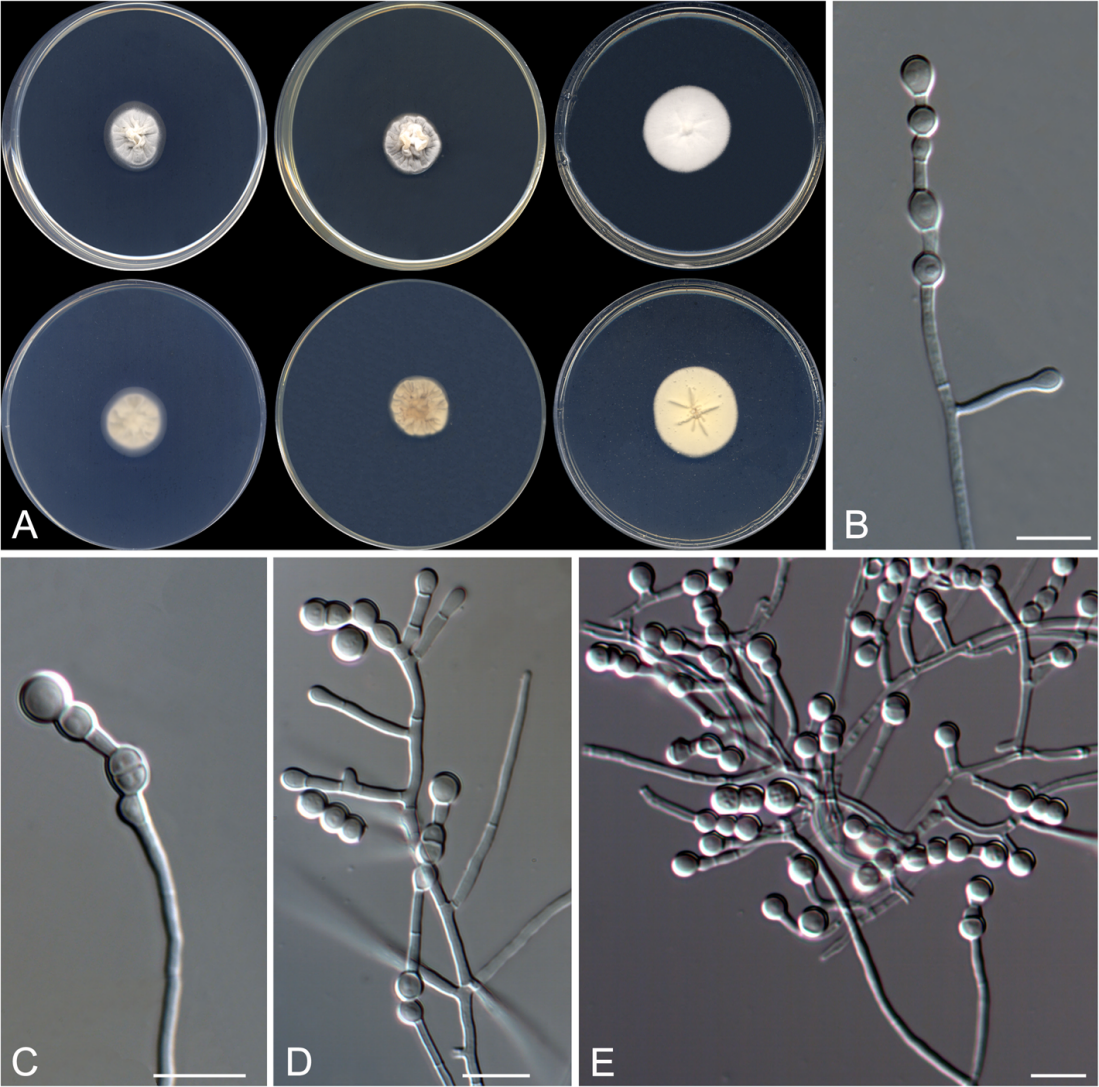
*Strongyloarthrosporum catenulatum* CBS 143841 ^T^. **a** Colonies on G18, G25 N and MY70FG at 25 °C (from left to right), surface and reverse (from top to bottom). **b**–**e** Conidiophores and conidia. Scale bar = 10 μm

Strongyloarthrosporum catenulatum Rodr.-Andr., Cano & Stchigel, sp. nov*.* Fig. 6. MycoBank MB 823588.

*Etymology:* From Latin *catenulatus*, in chains, referring to the disposition of the conidia.

*Diagnosis*: *Strongyloarthrosporum catenulatum* is phylogenetically close to the *Ajellomycetaceae*, a family of non-xerophilic fungi characterized by their thermally dimorphic nature and, consequently, pathogenic for animals. By contrast, *S. catenulatum* is an obligate xerophilic fungus with globose conidia sometimes disposed in chains.

*Type*: Spain: *Castilla-La Mancha community*: Toledo province, from decanted, filtered and thermally treated honey, 12 May 2014, *A. Terrab* (CBS H- 23371 – holotype; CBS 143841 = FMR 16121 – ex-type cultures; LSU sequence GenBank LT906534).

*Description: Colonies* on G18 reaching 20–21 mm diam after 3 wk. at 25 °C, elevated, velvety, sulcate, sporulation sparse, exudate absent, yellowish white (4A2) at the centre and white (3A1) at the edge; reverse orange-grey (5B2), diffusible pigment absent. *Mycelium* composed of hyaline, septate, smooth, thin- to thick-walled, anastomosing hyphae, 1.5–4 μm wide. *Conidiophores* reduced mostly to single fertile side branches and to the terminal part of the vegetative hyphae, 5–60 μm long, hyaline, disarticulating in conidia. *Conidia* hyaline, mostly one-celled, occasionally two-celled, holo- and enteroarthric, solitary, disposed terminally, intercalary or sessile on the fertile hyphae, or produced in basipetal chains of up to ten conidia, smooth-walled, thicker than the hyphae, thickener at the ends, mostly globose, 3–6 μm diam, flattened or not at one or both ends, disarticulating by rhexolytic secession from the conidiogenous hyphae. *Chlamydospores* and racquet hyphae absent.

*Colonies* on G25 N reaching 19–20 mm diam after 3 wk. at 25 °C, elevated, velvety, sulcate, exudate absent, sporulation sparse, light orange (5A4) at the centre and grey (5B1) at the edge; reverse greyish orange (5B5), diffusible pigment absent; on MY70FG reaching 29–30 mm diam after 3 wk. at 25 °C, flat, floccose, margins entire, sporulation sparse, white; reverse light yellow (4A4), diffusible pigments absent.

Minimum, optimal and maximum temperature of growth on G18 are 15 °C, 25 °C, and 35 °C, respectively, does not grow on blood agar, BCP-MS-G, Sabouraud dextrose agar with different NaCl concentrations, TOTM, OA, PYE nor on Christensen’s urea agar.

### Subclade B: ***Eurotiales***

Due to both LSU-based (Fig. 2; sister clade B1) and ITS-*BenA*-*CaM*-*rpb*2-based (Fig. 4) phylogenetetic trees, four of our *Talaromyces* strains were placed in section *Trachyspermi* in a well-supported subclade divided in two branches, and one more strain was placed into the section *Purpurei* in a basal position (Fig. 4), phylogenetically distant and phenotypically different from other species of *Talaromyces* in this section, consequenly, we propose the recognition of three new species of the genus.

**Fig. 7:**
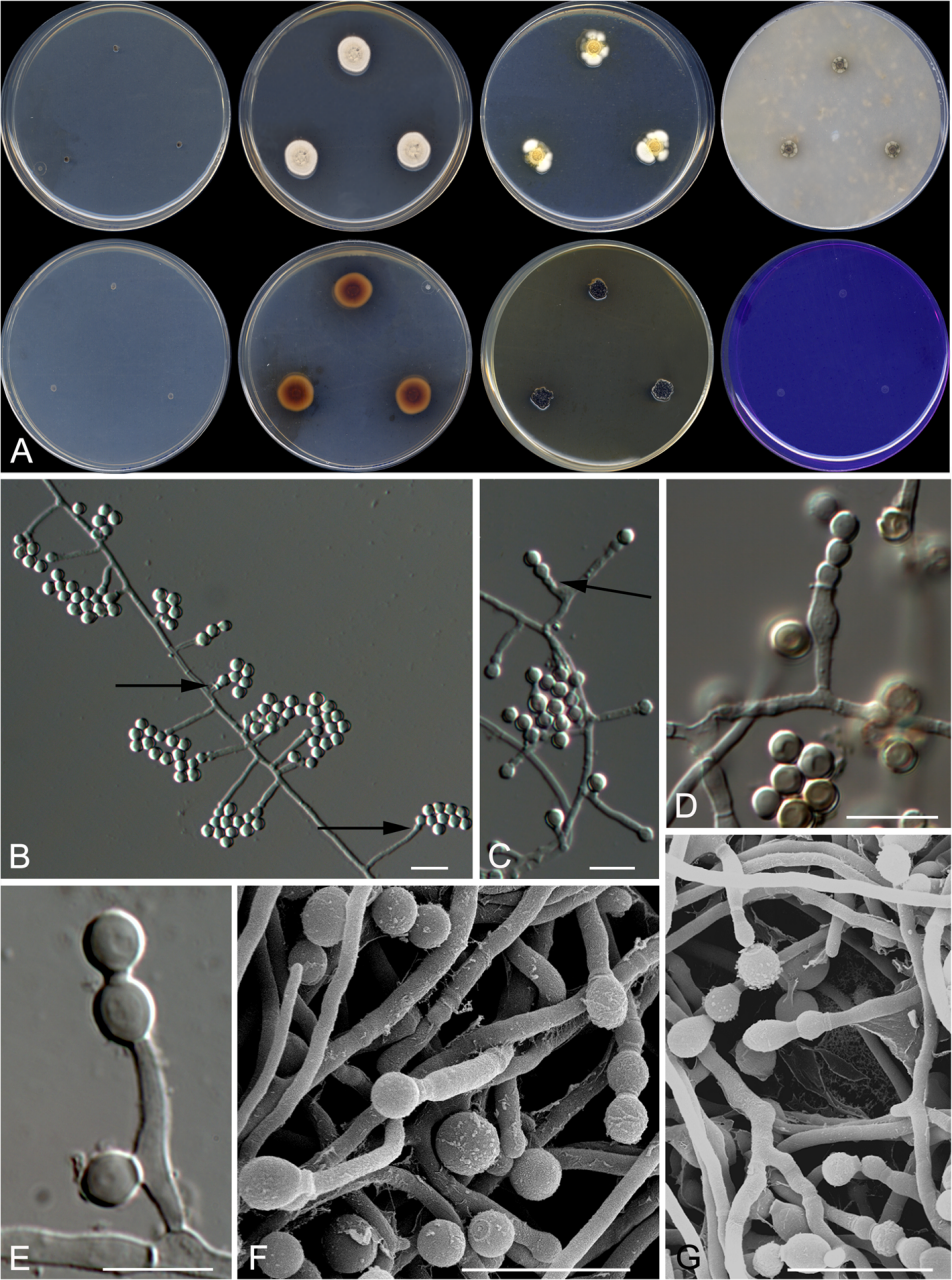
*Talaromyces basipetosporus* CBS 143836 ^T^. **a** Colonies from left to right (top row) CYA, MEA, DG18 and OA; (bottom row) CYA reverse, MEA reverse, YES and CREA. **b**–**g** Conidiophores and conidia; the arrows shows the conidia formed basipetally. Scale bar = 10 μm

Talaromyces basipetosporus Stchigel, Cano & Rodr.-Andr., sp. nov*.* Fig. 7. MycoBank MB 823589.

*Etymology*: After the morphological similarity to the asexual morph of *Basipetospora* (formerly applied to the asexual morph of *Monascus*).

*Diagnosis:* Differs from other species in sect. *Trachyspermi* in that the conidiogenesis is very similar to that of *Monascus* (syn. *Basipetospora*), characterized by retrogressively produced conidia, which have not been previously described in *Talaromyces* (see diagnosis of *Talaromyces affinitatimellis*).

*Type*: Argentina: *Buenos Aires province*: San Martín, from decanted, filtered and thermally treated honey, 1 Oct. 2007, *M. A. Álvarez* (CBS H-23365 – holotype; CBS 143836 = FMR 9720 – ex-type cultures; LSU sequence GenBank LT964940).

*Description*: *Colonies* on MEA reaching 10–11 mm diam after 3 wk. at 25 °C, slightly elevated, velvety to floccose, margins entire, yellowish grey (4B2) at the centre and white (4A1) at the edge, exudate absent, sporulation sparse; reverse brownish red (8C8) at the centre and greyish orange (5B6) at the edge, diffusible pigments absent. *Mycelium* abundant, composed of subhyaline to pale brown, smooth to echinulate, thin-walled, septate, anastomosing hyphae, of 2–3 μm wide. *Conidiophores* mostly reduced to a single conidiogenous cell, sometimes slender and with an additional conidiogenous locus near the base, arising alternately or oppositely at both sides of the vegetative hyphae, mostly separate from the vegetative hyphae by a basal septum. *Conidiogenous cells* smooth-walled to echinulate, mostly cylindrical and occasionally slightly slender towards the apex, sometimes broadening below the apex, but also flask- or barrel-shaped, very variable in length, 3–20(− 45) × 1–2.5 μm, conidiogenesis retrogressive. *Conidia* one-celled, hyaline and echinulate when young, becoming brown to dark brown and nearly smooth-walled with the age, formed basipetally, in false chains of up to ten conidia, mostly globose, 3.0–5.0 μm diam. Sexual morph not observed.

*Colonies* on DG18 reaching 13–14 mm diam after 3 wk. at 25 °C, colonies moderately elevated, texture floccose, yellowish orange (4B7) with mycelium white (5A1) at edge, sporulation dense, exudate absent, diffusible pigments absent, reverse reddish golden (6C7) at centre and pale yellow (3A4) at edge; on G18 reaching 10–11 mm diam after 3 wk. at 25 °C, slightly elevated, velvety to floccose, margins regular, yellowish white (3A2), exudates uncolored, diffusible pigment absent, reverse pale orange (5A3) at the centre and white at the edge; on OA reaching 5–6 mm diam. After 3 wk. at 25 °C, flat, margins entire, mycelium grey, texture velvety to floccose, sporulation dense, diffusible pigments absent, exudate absent, colonies dark brown (5D4) at centre and grey with olive-brown (6B1-4E6) patches at edge; on PDA reaching 10–11 mm diam. After 3 wk. at 25 °C, elevated, velvety, brown (7E7) at the centre and brownish grey (4D2) at the edge, sporulation abundant, exudate absent, diffusible blackish olive (2G6) pigment present, reverse dark brown (7F4) at centre and brown (7E8) at the edge; on YES reaching 7–8 mm diam after 3 wk. at 25 °C, moderately elevated, sulcate, rough, sporulation strong, blackish brown (6G8), diffusible pigments absent, exudates absent, reverse yellowish brown (5E8).

Minimum, optimal and maximum temperature of growth on G18 are 15, 25, and 30 °C, respectively; does not grow on CYA, Czapek 20% sucrose, CREA, Starch agar, or MY70FG.

Talaromyces brunneosporus Rodr.-Andr., Cano & Stchigel, sp. nov.

**Fig. 8:**
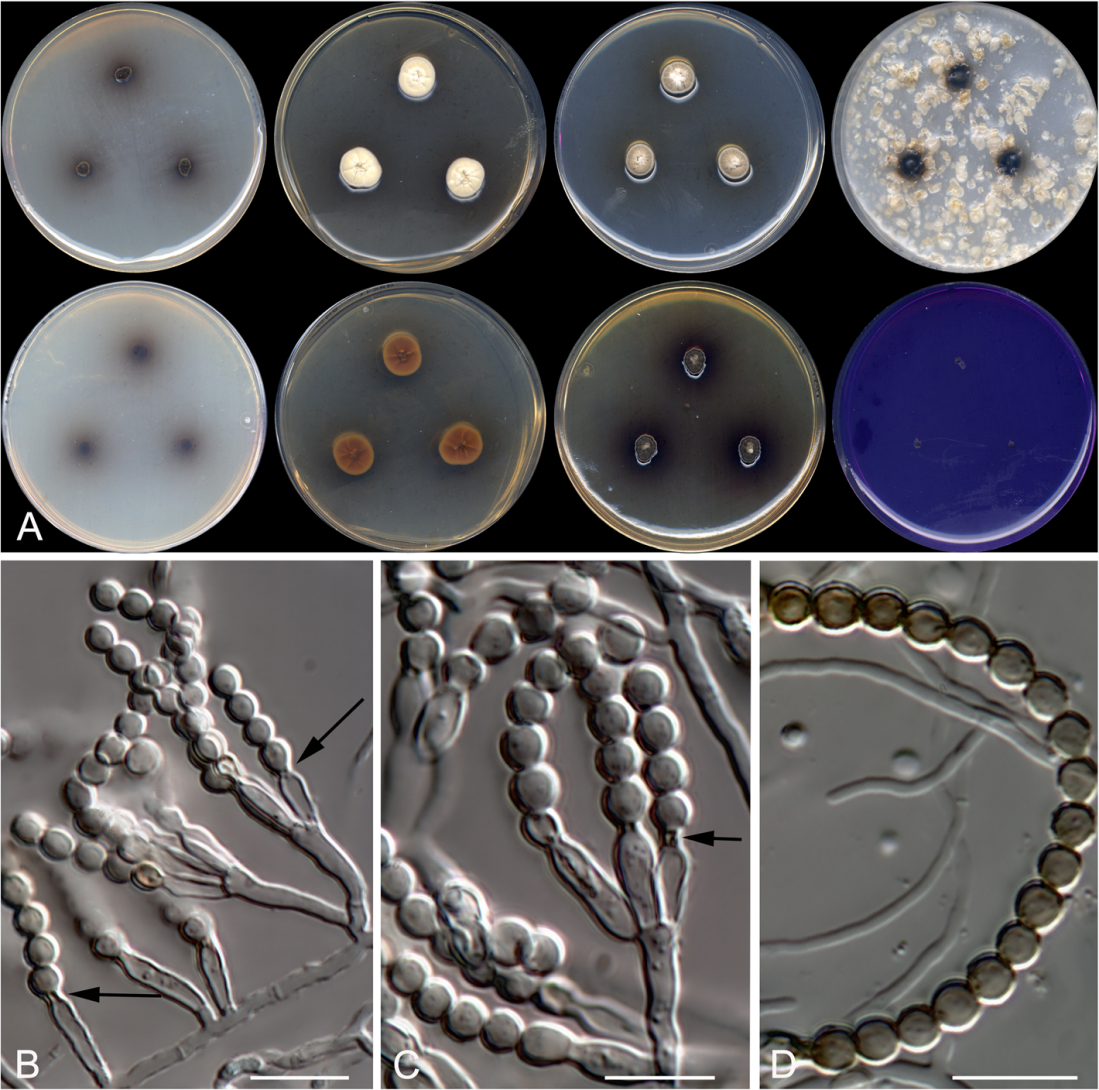
*Talaromyces brunneosporus* CBS 144320^T^. **a** Colonies from left to right (top row) CYA, MEA, DG18 and OA; (bottom row) CYA reverse, MEA reverse, YES and CREA. **b**, **c** Poorly-developed (single phialide) and well-developed (monoverticillate) conidiophores; the arrows indicate the conspicuous collarette at the top of the phialides. **d** A chain of globose, dark brown, verrucose conidia. Scale bar = 10 μm

Figure 8. MycoBank MB 823590.

*Etymology:* From Latin *brunneus*-, brown, and -*sporarum*, spore, in reference to the colour of the conidia.

*Diagnosis*: Distinguished from other species in sect. *Purpurei*, with the exception of *T. purpurei* (the type species of the section), by the production of solitary phialides and monoverticillate conidiophores (biverticillate conidiophores in the other species of the section). However, *T. brunneosporus* can be differentiated from *T. purpureus* because lack of a sexual morph (present in the latter species), and produces penicillate conidiophores (having an aspergillate look in *T. purpureus*) and verrucose conidia (ornamented with spiral ridges in *T. purpureus*).

*Type*: Spain: *Castilla y León community*: Salamanca province, from decanted, filtered and thermally treated honey, 1 Oct. 2014, *A. Terrab* (CBS H-23375 – holotype; CBS 144320 = FMR 16566 – ex-type cultures; LSU sequence GenBank LT964943).

*Description*: Colonies on MEA reaching 13–14 mm diam after 3 wk. at 25 °C, slightly elevated, velvety to floccose, margins irregular, yellowish white (4A3), exudate absent, sporulation sparse, reverse light brown (6D8) at the centre and yellowish brown (5D6) at the edge, diffusible yellowish brown (5E6) pigment present. *Mycelium* abundant, composed of subhyaline, smooth- and thin-walled, septate, anastomosing hyphae 2–3 μm wide. *Conidiophores* mostly stalked, monoverticillate, smooth- and thin-walled, bearing one to four conidiogenous cells at the top, frequently arising oppositely at both sides of the vegetative hyphae, sometimes reduced to a single conidiogenous cell, sessile or integrated to the vegetative hyphae (= adelophialides). *Conidiogenous cells* phialidic, smooth-walled, mostly slender towards the apex, flask-shaped, 8–12 × 2.5–3.5 μm, with a darkened apical area when the conidiogenous cells have produced several conidia, conidiogenesis enteroblastic. *Conidia* one-celled, globose, hyaline and smooth-walled when young, becoming brownish-green to dark brown and verrucose with the age, 3–4 μm diam, in long false chains of up to 25 conidia. *Sexual morph* not observed.

*Colonies* on CYA reaching 4–5 mm diam after 3 wk. at 25 °C, elevated, velvety, dark brown (8F4) at the centre and greyish-brown (7E3) at the edge, exudate absent, sporulation abundant, reverse dark brown (8F6) at the centre and reddish brown (8E5) at the edge, diffusible brown (6E7) pigment present; on DG18 reaching 10–11 mm diam after 3 wk. at 25 °C, moderately elevated, floccose, margins irregular, yellowish white (4A2) at the centre and olive-brown (4D6) at the edge, exudate absent, sporulation strong, reverse light brown (5D7), diffusible yellowish brown (5D5) soluble pigment present; on OA reaching 9–10 mm diam after 3 wk. at 25 °C, flat, floccose, margins entire, exudate absent, sporulation strong, colonies blackish olive (2G6) at the centre and brown (6E6) at the edge, diffusible olive brown (4E8) pigment present; on YES reaching 8–9 mm diam after 3 wk. at 25 °C, flat, floccose, black at the centre and yellowish-brown (5E6) at the edge, exudate absent, sporulation sparse, reverse dark violet (8E8), diffusible blackish brown (6G8) pigment present.

Minimum, optimal and maximum temperature of growth on G18 are 15, 25, and 30 °C, respectively; no growth on CYA at 37 °C nor on CREA at 25 °C.

*Notes: Talaromyces brunneosporus* and *T. purpureus* grow more slowly on CYA and MEA than other species of the section. However, *T. brunneosporus* produces dark brown colonies with a dark brown diffusible pigment on CYA, while the colonies of *T. purpureus* are pale beige and without diffusible pigments. Also, the colonies on OA and MEA are purplish in *T. purpureus* and pale coloured and dark brown in *T. brunneosporus*.

**Fig. 9:**
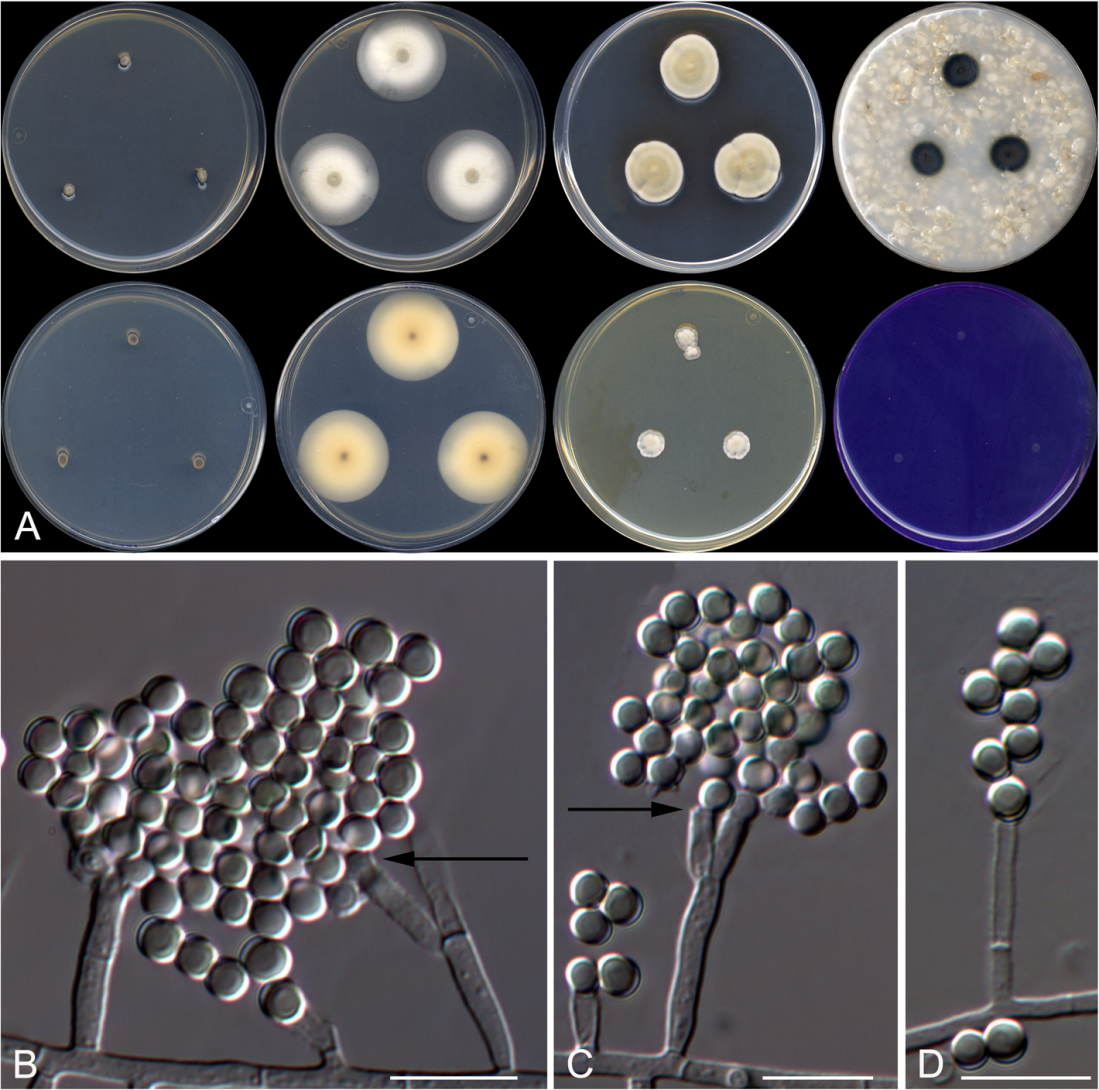
*Talaromyces affinitatimellis* CBS 143840 ^T^. **a** Colonies from left to right (top row) CYA, MEA, DG18 and OA; (bottom row) CYA reverse, MEA reverse, YES and CREA. **b**–**d** Conidiophores and conidia; the arrows shows the conidia formed basipetally. Scale bar = 10 μm

Talaromyces affinitatimellis Rodr.-Andr., Stchigel & Cano, sp. nov*.* Fig. 9. MycoBank MB 823591.

*Etymology*: From Latin *affinitatis*-, affinity, and -*mellis*, honey, after the substrate from which the fungus was isolated.

*Diagnosis*: Differing from all other species in sect. *Trachyspermi* (with the exception of *T. basipetosporus*) by the production of conidia by retrogressive conidiogenesis. *Talaromyces affinitatimellis* differs from *T. basipetosporus* by the production cylindrical, smooth-walled to echinulate conidiogenous cells ending in a greenish brown, broad collarette-like structure (conidiogenous cells irregularly-shaped, smooth-walled, and without such apical structure in *T. basipetosporus*).

*Type*: Spain: *Valencia community*: Castellón province, from decanted and filtered blossom honey, 10 May 2014, *A. Gómez Pajuelo* (CBS H- 23370 – holotype; CBS 143840 = FMR 15690 – ex-type cultures; LSU sequence GenBank LT964939).

*Description*: Colonies on MEA reaching 29–30 mm diam. After 3 wk. at 25 °C, flat, floccose, not sulcate, margins entire, olive (3D3) at the centre and white (4A1) at edge, exudate absent, sporulation sparse; reverse pale orange (5A3) at centre and pale yellow (4A3) at edge, diffusible pigment absent. *Mycelium* abundant, composed of subhyaline to pale brown, smooth- and thin-walled, septate, anastomosing hyphae, of 2–4 μm wide. *Conidiophores* hyaline to pale brown, reduced to a single conidiogenous cell, occasionally with an additional conidiogenous locus near the base or lateraly disposed, or short-stalked and bearing two conidiogenous cells, sometimes with an additional lateral conidiogenous cell arising alternately at both sides of the vegetative hyphae, separate from them by a basal septum. *Conidiogenous cells* hyaline to pale brown, smooth-walled, mostly cylindrical and occasionally slightly slender towards the apex, sometimes ending in a greenish-brown, broad collarette-like structure, 3–20 × 1.5–3 μm, conidiogenesis retrogressive but enteroblastic. *Conidia* one-celled, hyaline and echinulate, becoming brown to dark brown and nearly smooth-walled with the age, produced basipetally in false chains of up to ten in number, mostly globose, 3.0–5.0 μm diam. Sexual morph not observed.

*Colonies* on DG18 reaching 13–14 mm diam after 3 wk. at 25 °C, moderately elevated, floccose, yellowish orange (4B7) with white (5A1) margins, exudates absent, sporulation strong; reverse reddish golden (6C7) at the centre and pale yellow (A4) at the edge, diffusible pigment absent; on G18 reaching 21–24 mm diam at 25 °C, slightly elevated, velvety to floccose, margins regular, yellowish white (4A4), exudates absent, sporulation abundant, reverse greyish orange (5B6), diffusible pigment absent; on OA reaching 12–13 mm diam after 3 wk. at 25 °C, flat, velvety to floccose, margins entire, black, exudates absent, sporulation abundant; colonies grey (7F1) at the centre and dark brown (6F4) to black at the edge, diffusible pigment absent; on PDA reaching 39–43 mm diam after 3 wk. at 25 °C, flat, velvety, margins slightly irregular, yellowish-brown (5F6) at the centre, grey (7F1) and yellowish brown (5E4) at the middle part, and light grey (5B1) at the edge, exudate absent, sporulation scarce, reverse dark brown (7F7) at the centre and brownish yellow (5C7) at the edge, diffusible pigment absent; on YES reaching 10–11 mm diam after 3 wk. at 25 °C, moderately elevated, floccose, white (4A1), exudate absent, sporulation sparse, reverse greyish orange (5B6), diffusible pigment absent.

Minimum, optimal and maximum temperature of growth on G18 are 15, 25, and 35 °C, respectively; no growth on CYA, Czapek 20% or CREA, or at 40 °C on all tested media.

*Other specimens examined:* Spain: *Catalonia community*: Tarragona province, from decanted and filtered blossom honey, 10 May 2014, *A. Gómez Pajuelo* (FMR 15674, FMR 15675, and FMR 15677); *Valencia community*: Castellón province, from decanted and filtered blossom honey, 10 May 2014, *A. Gómez Pajuelo* (FMR 15684 and FMR 15688); *Extremadura community*: Cáceres province, from decanted, filtered and thermally treated honeydew honey, 16 May 2014, *A. Terrab* (FMR 16029, FMR 16499, and FMR 16501); *Castilla y León community*: Salamanca province, from decanted, filtered and thermally treated honeydew honey, 01 Oct. 2014, *A. Terrab* (FMR 16033 and FMR 16114); Zamora province, from decanted, filtered and thermally treated honeydew honey, 05 Oct 2014, *A. Terrab* (FMR 16125, FMR 16126, FMR 16276, and FMR 16494).

### Subclade D: *Incertae sedis*

Based on both LSU-based (Fig. 2; sister clade D1) and ITS-based (Fig. 3) phylogenetic trees, ten of our strains were located in a well-supported and separated branch related to species of the genera *Oidiodendron* and *Myxotrichum*, and phylogenetically distant from the most similar taxa included in the study, *M. setosum* and *O. truncatum* (Fig. 3). Recognition of all of these distinct strains was also supported by unique phenotypic characteristics; therefore, we propose the recognition of the new species *Oidiodendron mellicola*. Furthermore, because three of our strains were placed near *Skoua fertilis* in both LSU-based (Fig. 2; sister clade D2) and ITS-based (Fig. 3) phylogenies and because they showed different phenotypic features and enough phylogenetic distance relative to *S. fertilis*, we also propose the introduction of a further new species, *Skoua asexualis*.

**Fig. 10:**
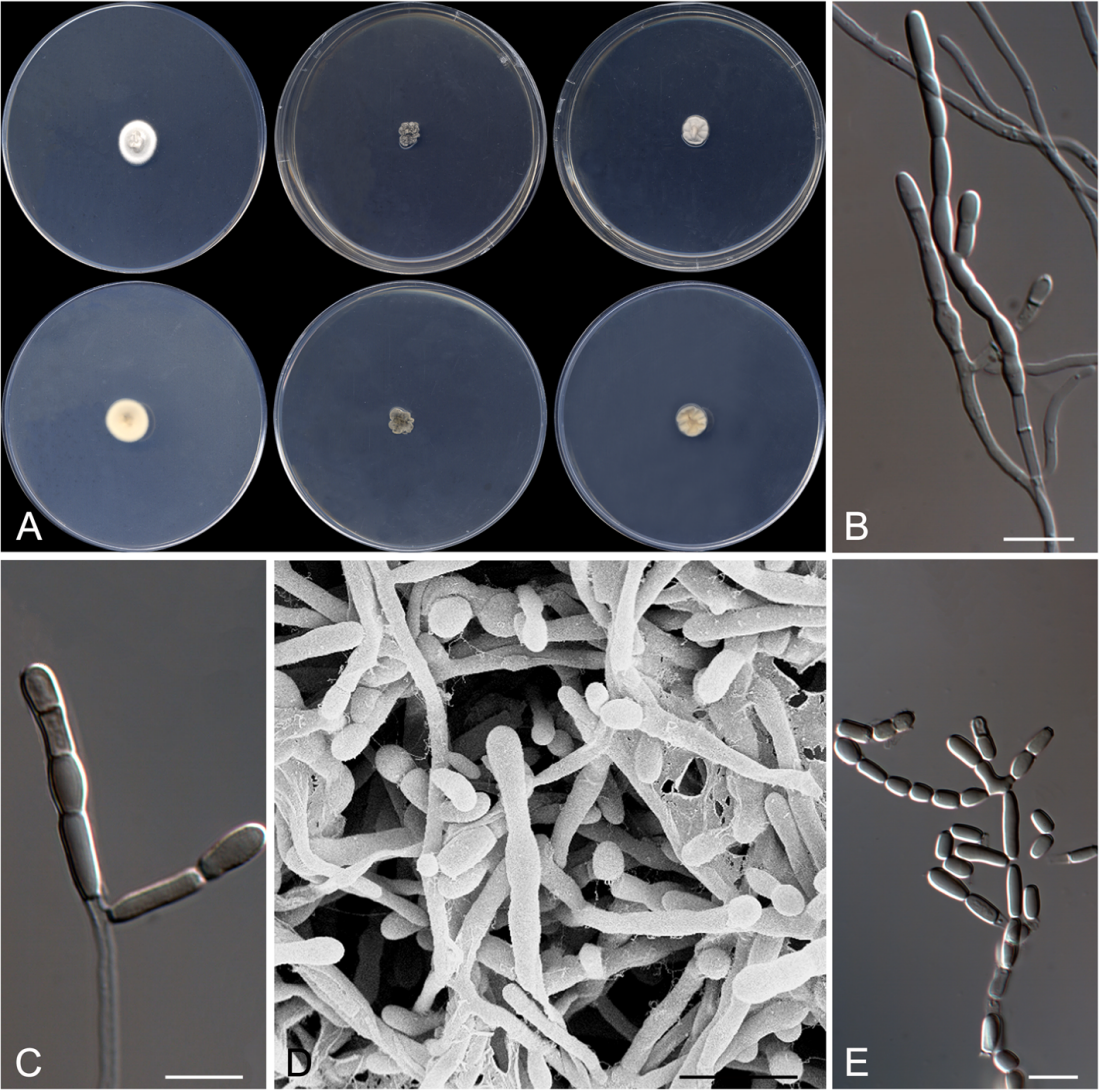
*Oidiodendron mellicola* CBS 143839 ^T^. **a** Colonies on PDA at 15 °C and at 25 °C, and on G18 at 25 °C (left to right), surface and reverse (from top to bottom). **b**–**d** Conidiophores. **e** Disarticulating chains of conidia. Scale bar = 10 μm

Oidiodendron mellicola Rodr.-Andr., Cano & Stchigel, sp. nov. Fig. 10. MycoBank MB 823586.

*Etymology*: From Latin *mellis*-, honey, and -*cola* dwelling on, referring to the habitat.

*Diagnosis*: Forming a terminal clade together with *O. truncatum* and *M. setosum* at a significant phylogenetic distance (5.3% from the other two species), and differing morphologically from other known species of *Oidiodendron* and the asexual morphs of *Myxotrichum* in the absence of well-differentiated conidiophores, and the slow growth.

*Type:* Spain: *Valencia community*: Castellón province, from decanted and filtered blossom honey, 10 May 2014, *A. Gómez Pajuelo* (CBS H-23369 – holotype; CBS 143839 = FMR 15683 – ex-type cultures; ITS sequence GenBank LT906544).

*Description*: *Colonies* on PDA at 15 °C reaching 15–16 mm diam after 3 wk., white (5A1), sporulation sparse (seen after 6 wk. of incubation), exudate absent, reverse orange-white (6A2) at the centre and orange-grey (6B2) at the edge, diffusible pigment absent. *Mycelium* composed of hyaline, septate, smooth- and thin-walled hyphae, 1–3 μm wide. *Conidiophores* reduced to fertile side branches and the terminal part of a vegetative hyphae, mostly simple or once branched near or at the base, 10–40 μm long, pale olive, disarticulating in conidia. *Conidia* one-celled, mostly holoarthric, sometimes enteroarthric, mostly in chains of up to ten, occasionally solitary and sessile, mostly barrel-shaped, sometimes cylindrical, conical or “Y”-shaped, 5–14 × 2.5–5 μm, pale olive, disarticulating by schizolytic or rhexolytic secession from the hyphae. *Chlamydospores* absent. *Sexual morph* absent.

*Colonies* on PDA reaching 10–11 mm diam. After 3 wk. at 25 °C, elevated, compact, velvety, margins irregular, olive brown (4E3), exudates absent, sporulation abundant; reverse olive brown (4E5) at the center, grey (5D1) at the edge, diffusible pigment absent. Colonies on G18 reaching 11–12 mm diam after 3 wk. at 25 °C, elevated, velvety to floccose, yellowish white (4A2) at the centre and white (4A1) at the edge, margins regular, sporulation absent, reverse pale yellow (4A3), diffusible pigment absent; on G18 at 15 °C reaching 12–15 mm diam after 3 wk., similar in aspect than at 25 °C; on MY70FG and MEA 2% at 25 °C after 3 wk. reaching 1–3 mm diam.

Minimum, optimal and maximum temperature of growth on G18 are 5, 15, and 25 °C, respectively; no growth on OA or PCA at 25 °C.

*Other specimens examined*: Spain: *Catalonia community*: Tarragona province, from decanted and filtered blossom honey, 10 May 2014, *A. Gómez Pajuelo* (FMR 15680); *Castilla-La Mancha community*, Ciudad Real province, from decanted, filtered and thermally treated honeydew honey, 10 May 2014, *A. Terrab* (FMR 16031, FMR 16117, and FMR 16503); Toledo province, from decanted, filtered and thermally treated honeydew honey, 12 May 2014, *A. Terrab* (FMR 16120 and FMR 16282); *Galicia community*: Ourense province, from decanted, filtered and thermally treated honeydew honey, 03 May 2014, *A. Terrab* (FMR 16504); *Castilla y León community*: Salamanca province, from decanted, filtered and thermally treated honeydew honey, 01 Oct. 2014, *A. Terrab* (FMR 16023); Burgos province, from decanted, filtered and thermally treated honeydew honey, 23 May 2014, *A. Terrab* (FMR 16274).

**Fig. 11:**
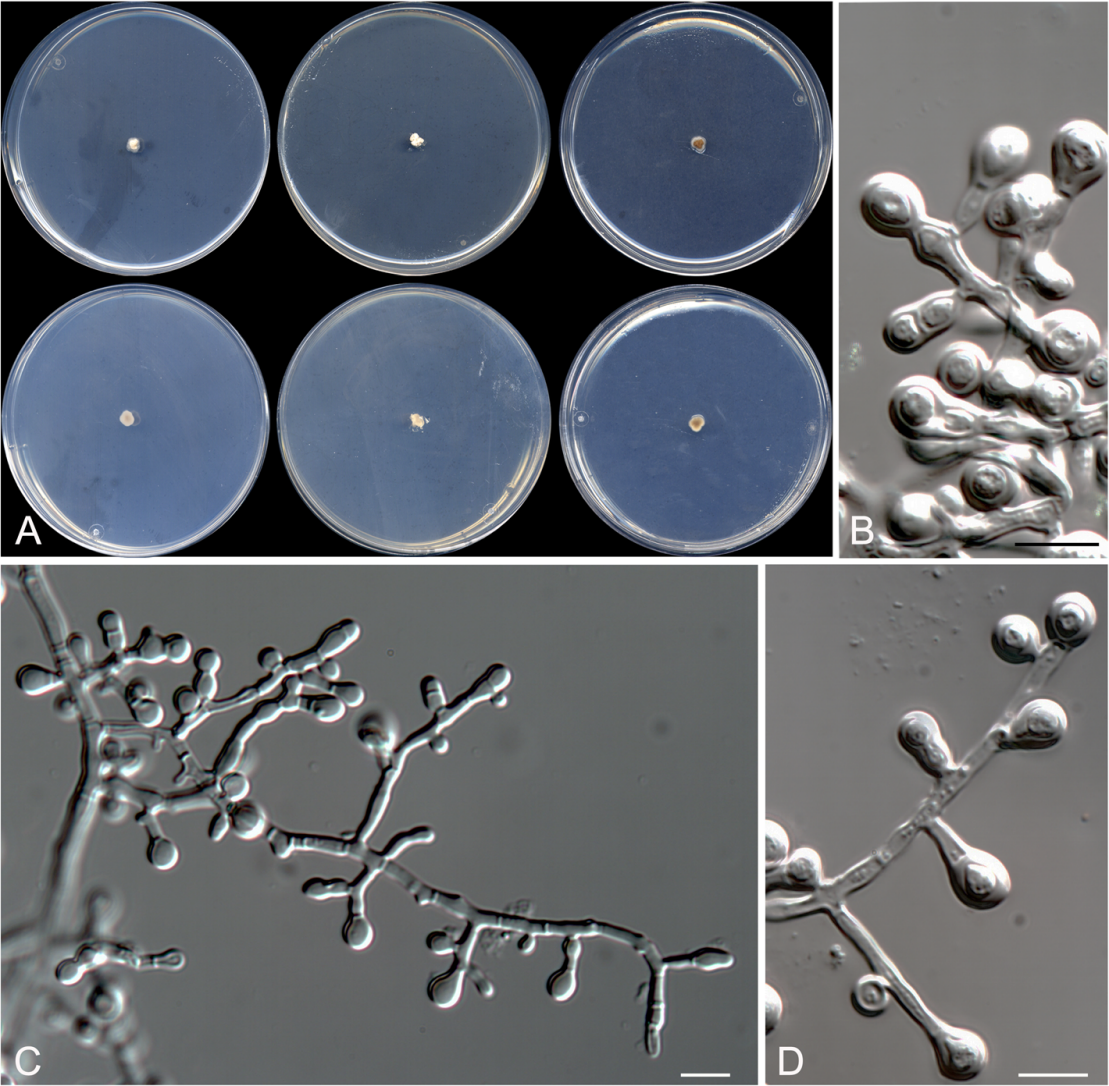
*Skoua asexualis* CBS 144072 ^T^. **a** Colonies on G18, MEA and PDA at 25 °C (left to right), surface and reverse (from top to bottom). **b**–**d** Conidiophores and conidia. Scale bar = 10 μm

Skoua asexualis Rodr.-Andr., Cano & Stchigel, sp. nov. Fig. 11. MycoBank MB 824092.

*Etymology*: From Latin *asexualis*, without sex, because of lack of a known sexual morph.

*Diagnosis*: Differing from the other known species of the genus, *S. fertilis*, in asexual reproduction, as the latter only produces ascospores within globose asci arising from the mycelium.

*Type:* Spain: *Castilla y León community*: León province, from decanted, filtered and thermally treated honeydew honey, 1 Oct. 2014, *A. Terrab* (CBS H-23397 – holotype; CBS 144072 = FMR 16572 – ex-type cultures; ITS sequence GenBank LT964668).

*Description*: *Colonies* on PDA reaching 6–7 mm diam after 3 wk. at 25 °C, elevated, velvety, sporulation abundant, exudates absent, diffusible pigment absent, colonies brown (7E6) at the centre and whitish at the edge, reverse brownish orange (6C5) at the centre and greyish orange (5B3) at the edge. *Mycelium* composed of hyaline, repeatedly septate, smooth- and thin-walled hyphae, 2–6 μm wide. *Conidiophores* absent. *Conidia* mostly one-celled, occasionally two- to three-celled, hyaline, solitary or in short chains, smooth- and thick-walled, mostly globose, occasionally broadly ellipsoidal, pyriform, or irregular-shaped, truncate at one or both ends, 3–7 μm diam, conidiogenesis holoblastic when sessile or terminal, and holothallic when intercalary, disarticulating by rhexolytic secession; the holoblastic and holothallic conidia produce a succession of secondary holoblastic conidia, forming a big, radiating mass of cells of up to 50 μm diam, which eventually detach as complex asexual propagules from the fertile hyphae. *Chlamydospores* similar to the conidia but thicker, mostly non- or occasionally one-septate, intercalary or terminal. *Sexual morph* unknown.

*Colonies* on MEA reaching 3–4 mm diam after 3 wk. at 25 °C, colonies elevated, velvety to floccose, margins irregular, sporulation abundant, diffusible pigment absent, mycelium yellowish white (4A2), reverse pale yellow (4A3); on G18 reaching 4–5 mm diam after 3 wk. at 25 °C, elevated, floccose, margins irregular, sporulation sparse, diffusible pigment absent, exudates absent, colonies pale yellow (4A3) at the centre, reverse orange-grey (5B2).

Minimum, optimal and maximum temperature of growth on G18 are 15, 25, and 30 °C, respectively; no growth on CYA, CREA, OA, or YES at 25 °C.

*Other specimens examined*: Spain: *Extremadura community*: Cáceres province, from decanted, filtered and thermally treated honeydew honey, 16 May 2014, *A. Terrab* (FMR 16497 and FMR 16567).

## DISCUSSION

**Fig. 12:**
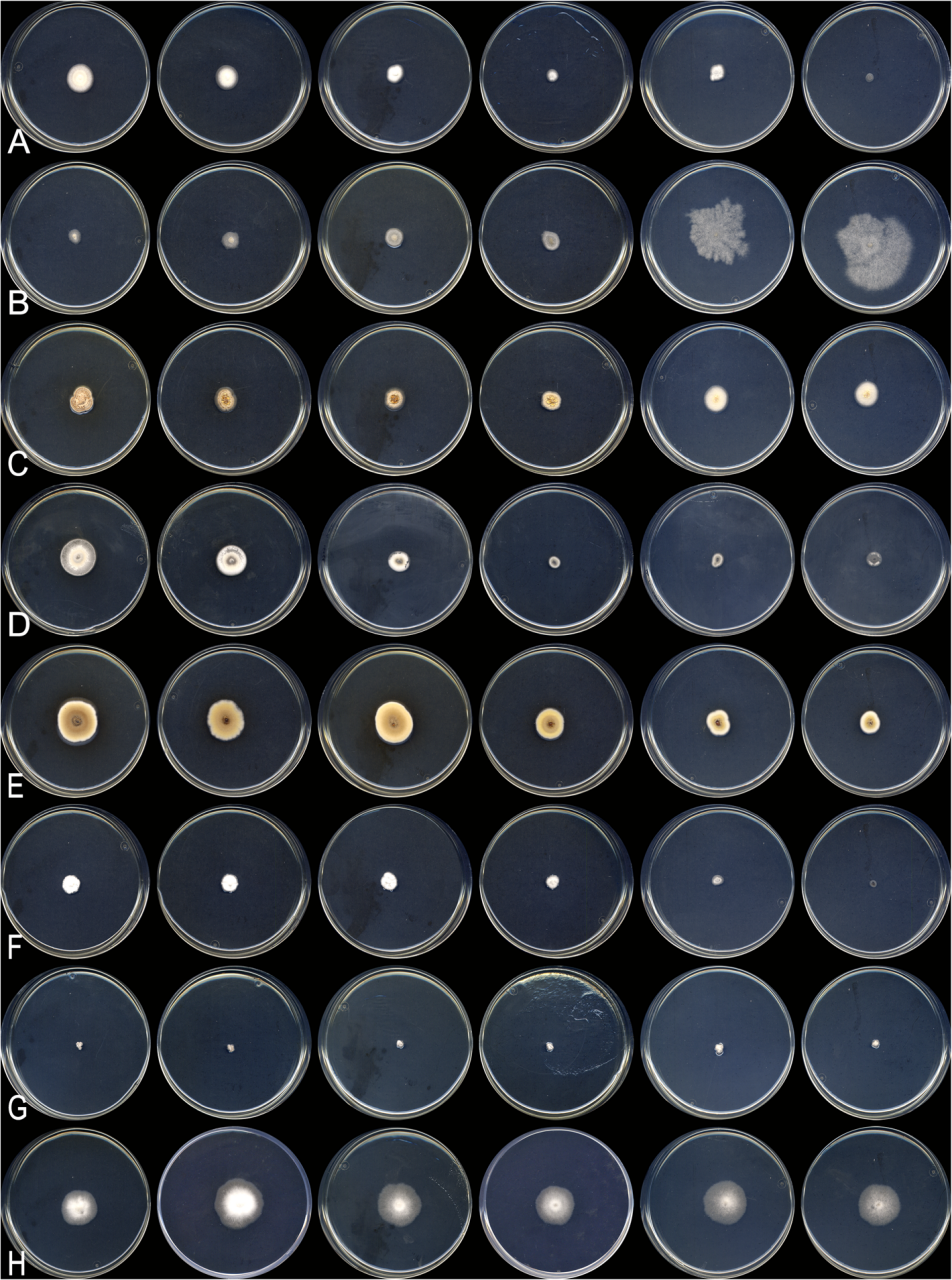
Relatedness between the growth of the new fungal taxa and the decreasing water activity (a_w_) of the culture medium. Surface of the colonies grown on MEA with a_w_ values of 0.97, 0.95, 0.93, 0.92, 0.88 and 0.82 (from left to right, respectively). **a** *Helicoarthrosporum mellicola*. **b** *Strongyloarthrosporum catenulatum*. **c** *Talaromyces basipetosporus*. **d** *Talaromyces brunneosporus.* **e** *Talaromyces affinitatimellis*. **f** *Oidiodendron mellicola*. **g** *Skoua asexualis.* **h** *Skoua fertilis* (as reference, highly xerotolerant fungus)

This is the most comprehensive assessment of the diversity of the xerotolerant and xerophilic fungi of honey intended for human consumption to date. We have isolated selectively and identified, by a polyphasic approach, six species of ascomycetous yeasts and 27 of filamentous ascomycetes, some representing new taxa, from honey samples. The yeasts, *Candida magnoliae*, *C. sorbosivorans*, *Schizosaccharomyces octosporus*, *Zygosaccharomyces barkeri*, Z. *mellis*, and *Z. gambellarensis*, had been reported from honey before, and *C. magnoliae* has also been associated with living honeybees (Gilliam et al. 1974b). All these yeasts have been described as osmophilic and able to grow at a_w_ of 0.80 or lower (Tilbury 1967; van Eck et al. 1993; Ganthala et al. 1994; Erickson & McKenna 1999; Torriani et al. 2011). We found *C. magnoliae* and *C. sorbosivorans* were phylogenetically closely related (see Fig. 2), and it was reported that both differ only in a few physiological characteristics (James et al. 2001). To our knowledge, none of the species of *Aspergillus* that we isolated (*A. asperescens*, *A. montevidensis*, and *A. pseudoglaucus*) have previously been reported from honey. *Aspergillus asperescens* was originally isolated from soil and bat dung (Stolk 1954), but also from rotten wood and soybean seeds; however, most of the isolates were from cave soil (probably linked to bat dung). *Aspergillus montevidensis* and *A. pseudoglaucus* have been reported as the most important food-spoilage species of the genus (Pitt & Hocking 1977; Kozakiewicz 1989), but are known from extreme environments such as salterns (Butinar et al. 2005). *Aspergillus montevidensis* has been reported from various environmental samples (air, soil, etc.), and even on honeybees and bee larvae (http://gcm.wfcc.info/; Talice & Mackinnon 1931; Gilliam et al. 1974a); *A. pseudoglaucus* has been reported in air, paper and soil (http://gcm.wfcc.info/; Blochwitz 1929). *Aspergillus montevidensis* and *A. pseudoglaucus* are able to grow at a_w_ values of 0.80 (Snow 1949; Armolik & Dickson 1956; Guynot et al. 2003). *Monascus* is a well-known genus with species (especially *M. purpureus* and *M. ruber*) of economic importance due to their use in production of foodstuffs, bioactive compounds, pigments and enzymes. Currently, *Monascus* is placed in *Aspergillaceae* (syn. *Trichocomaceae*) based on phylogenetic studies, and closely related to *Leiothecium ellipsoideum* and *Xeromyces bisporus* (Houbraken & Samson 2011; Pettersson et al. 2011). Recently, three new species were added, all of them associated with stingless bees: *M. flavipigmentosus*, *M. mellicola,* and *M. recifensis* (Barbosa et al. 2017). We found a small number of isolates, including *M. pilosus*, *M. purpureus,* and *M. ruber*. These species have been frequently reported in fermented and spoiled foods (van Tieghem 1884; Hesseltine 1965; Lin 1975; Hawksworth & Pitt 1983). *Monascus ruber* has also been found in soil and human clinical specimens (Hawksworth & Pitt 1983). Species of *Monascus* have been previously reported in honey by Snowdon & Cliver (1996) and by Barbosa et al. (2017). *Monascus pilosus*, *M. purpureus,* and *M. ruber* were reported previously (Hawksworth & Pitt 1983) as able to grow well on G25 N (a_w_ = 0.93). The species of *Penicillium* we found in honey included *P. camemberti*, *P. citrinum, P. corylophilum,* and *P. cravenianum*. The most common source of isolation of *P. camemberti* is blue cheeses, but it can also be found on a wide variety of substrata (Thom 1906; http://gcm.wfcc.info/). *Penicillium citrinum* was originally reported in milk and bread in the USA (Thom 1910), but it is found globally and easy to recover from spoiled foods and diverse environmental sources (www.cabri.org/collections.html) including honey, pollen and bee nests (Barbosa et al. 2018). *Penicillium corylophilum* (Dierckx 1901) mostly occurs in damp buildings in North America and Western Europe, but also in foods and mosquitoes (Da Costa & De Oliveira 1998; McMullin et al. 2014), and honey (Sinacori et al. 2014). The minimum a_w_ reported for the growth of *P. camemberti*, *P. citrinum* and *P. corylophilum* was around 0.80 (Abellana et al. 2001; Fontana 2008; Kalai et al. 2017). *Penicillium cravenianum*, a species moderately xerotolerant (grows on G25 N), has only been reported in soil (Visagie et al. 2016). Notably, all the isolates of *Talaromyces* that we found in honey belonged to three unrecognized species. *Talaromyces basipetosporus* was recovered from a honey sample in Buenos Aires province, Argentina, and is characterized by simple conidiophores that mimic those of the asexual morph of *Monascus* (syn. *Basipetospora*), which develops conidia by a retrogressive mode of conidiogenesis, a feature not previously reported in *Talaromyces*. *Talaromyces affinitatimellis* displays a similar conidiogenesis to *T. basipetosporus* and both species are phylogenetically closely related but phenotypically differentiated as *T. affinitatimellis* grows faster and produces more complex conidiophores. *Talaromyces brunneosporus* differs from the other species of sect. *Purpurei*, apart from *T. purpureus*, in having monophialidic and monoverticillate conidiophores (they are biverticillate in the other species). However, both species are distinguishable because *T. brunneosporus* produces penicillate conidiophores (not aspergillate as in *T. purpureus*), longer phialides, and verrucose conidia with a flattened base (*T. purpureus* conidia are ornamented by spiral ridges). *Talaromyces basipetosporus* has a high xerotolerance, with similar growth rates on MEA with sugars up to a_w_ 0.82. Despite the decreasing growth rates of *T. brunneosporus* and *T. affinitatimellis* when sugar concentration increases, both fungi are able to grow at a_w_ 0.82 (Fig. 12). *Xerochrysium xerophilum* (Pitt et al. 2013; syn. *Chrysosporium xerophilum*, Pitt 1966), is an extreme xerophile with a minimum a_w_ for growth of 0.66 (Gock et al. 2003; Leong et al. 2011). This fungus, previously reported from chocolate, coconut, dried prunes, and stored corn (Pitt & Hocking 2009; Pitt et al. 2013), has not been found in honey until now. This species is phylogenetically close to *Monascus* (Pitt et al. 2013). Among the species of *Onygenales*, *Ascosphaera atra* and *Eremascus albus* were recovered once and four times, respectively. *Ascosphaera atra* (Skou & Hackett 1979) was originally reported from dead larvae of the alfalfa leafcutter bee covered in cysts of *Ascosphaera aggregata* (Skou 1975), and from pollen in the gut of healthy leafcutter larvae. This fungus was subsequently reported from grass silage (Skou 1986). *Ascosphaera atra* is homothallic and saprobic, probably being a common contaminant of pollen (Skou & Hackett 1979), which would explain its presence in honey samples. *Eremascus albus* is a well-known xerophilic fungus, with spores that can germinate at a_w_ as low as 0.70 (Pitt 1965). This fungus has been reported to spoil malt extract (Eidam 1883), chocolate cake, dried fruits, and mustard powder (Harrold 1950), but never previously from honey. We identified several isolates belonging to the newly described family *Helicoarthrosporaceae*, which only includes the new monotypic genus *Helicoarthrosporum*, and a single strain belonging to the new monotypic genus *Strongyloarthrosporum* (*Ajellomycetaceae*). The morphology of *Helicoarthrosporum mellicola* resembles species of *Scytalidium* (*S. cuboideum*, *S. ganodermophthorum,* and *S. sphaerosporum*) because of the production of cuboid arthroconidia in long chains. However, *Helicoarthrosporum* is phylogenetically distant from *Scytalidium*, as the latter is related to *Myxotrichaceae*. *Strongyloarthrosporum catenulatum* was found to be phylogenetically close to *Ajellomycetaceae*, whose members are thermally dimorphic and pathogenic to animals (including the humans), and has never been reported as xerotolerant. However, having features not seen in that family, *S. catenulatum* is unequivocally a xerophilic fungus, only growing on G18, G25 N and MY70FG, and producing globose arthroconidia, either singly or in chains. The sole xerophilic fungus phylogenetically close to *S. catenulatum* is *Eremascus albus* (*Eremascaceae*), but it only develops a sexual morph. Regarding the family *Myxotrichaceae*, *Skoua fertilis*, which was detected in all honey samples, resembles *Eremascus albus* (Eidam 1883) in having naked asci arising directly out of the mycelium and formed by the fusion of two equal cells borne on short entwined hyphae. Both taxa can be only morphologically differentiated by the shape of the ascospores and by sexual reproductive details. While *S. fertilis* (syn. *E. fertilis*) belongs to *Leotiomycetes*, closely related to *Myxotrichaceae* (Wynns 2015), *E. albus* is located in *Eurotiomycetes*, closely related to *Onygenales* (Cai et al. 1996; Berbee 2001; Wynns 2015). *Skoua* was introduced for *E. fertilis* (i.e. *Skoua fertilis*) and has been reported on bee bread, honeycomb, dried prunes and spoiled moist prunes, green compost, and shortcake (www.cabri.org/collections.html; http://gcm.wfcc.info/; Harrold 1950), but not so far on honey. The minimum a_w_ for growth and sporulation reported for *S. fertilis* was 0.77 (Pitt 1965; Wynns 2015), a similar value observed in all our strains (0.82). We isolated three strains of *Skoua* phylogenetically different from *S. fertilis*, and named them as *Skoua asexualis* because they form asexual spores instead of the sexual spores as observed in the type species of the genus. *Bettsia alvei* (Skou 1972, 1975), the other fungus identified in all honey samples, belongs to *Pseudeurotiaceae* and is characterized by dark, closed ascomata (usually called “spore cysts”) and hyaline globose ascospores, forming a sticky mass. *Bettsia alvei* has been isolated from hives in Europe as well as the USA (Burnside 1929), and from bakery products, spoiled chocolate, desiccated coconut, honeycomb, concentrated jelly, dried and spoiled prunes, pollen, table jelly, bee wax, and wine starters (www.cabri.org/collections.html; http://gcm.wfcc.info/). It was also isolated from chocolate in Austria (a_w_ less than 0.3), but thus far had not been recorded from honey. The lowest a_w_ tested for growth of this species was 0.88 (Beuchat & Pitt 1990) and 0.89 (Udagawa & Toyazaki 2000), similar values to those we found. All our isolates of *B. alvei* developed the chrysosporium-like asexual morph but failed in the production of the sexual morph. Among the most frequent species we isolated was an undescribed species of *Oidiodendron*, *O. mellicola*. Species of this genus are mostly recovered from soil and other substrata rich in cellulose, and are found worldwide (Domsch et al. 1980; Calduch et al. 2004; Rice & Currah 2005). *Oidiodendron mellicola* is phylogenetically related to *O. truncatum* and *M. setosum,* the former characterized by well-differentiated dark conidiophores and barrel-shaped conidia with a dark scar at one or both ends (typical features of *Oidiodendron*), and the latter by hyaline conidiophores and conidia, and by dark brown to black, spinose, gymnothecial ascomata (typical of the genus *Myxotrichum*). Interestingly, *M. setosum* is reported as a common hive fungus in Europe (Burnside 1929). *Oidiodendron mellicola* is the only species of the genus reported from honey, and it can be distinguished morphologically from other species of the genus by its absence of stipitate conidiophores, and the production of long chains of conidia, which are pale, smooth, ellipsoidal to cylindrical, truncated (but not darkened, as in *O. truncatum*) at one or both ends, and by the slow growing colonies. Like most of the species of the genus, *O. mellicola* grows better at 15 °C than 25 °C. Other fungi rarely found in our study were *Alternaria multiformis*, previously only reported from soil (Simmons 1998), and the mucoralean *Cunninghamella bertholletiae*, *Mucor plumbeus,* and *Rhizopus oryzae*, all found worldwide. These probably represent environmental contaminants. Although all the new taxa that we propose displayed a high xerotolerance, only *Strongyloarthrosporum catenulatum* can be considered an obligate xerophile, because it was able to grow faster at the lowest a_w_ tested (Fig. 12).

## CONCLUSION

The application of G18 as a selective culture medium for isolation of xerotolerant/xerophilic fungi from honey samples enabled the recovery and identification of 13 genera and 29 species of *Ascomycota*, and three genera (one species for each) of *Mucoromycota*. Many of these fungi have never reported from honey before. Among them, we proposed a new family (*Helicoarthrosporaceae*), two new genera (*Strongyloarthrosporum* and *Helicoarthrosporum*) and seven new species (*Strongyloarthrosporum catenulatum*, *Helicoarthrosporum mellicola*, *Oidiodendron mellicola*, *Skoua asexualis Talaromyces basipetosporus*, *T. brunneosporus*, and *T. affinitatimellis*). All fungal taxa that we isolated from honey were able to grow at low water activity (up to 0.82), but only *Ascosphaera atra*, *Bettsia alvei* (two fungi strongly associated to honeybees and their life-style), *Eremascus albus*, *Strongyloarthrosporum catenulatum* (one of the new taxa we described) and *Xerochrysium xerophylum* can be considered obligate xerophiles. Also, because several of the honey samples were thermally treated, these fungi can be considered as hot-resistant. Honey is evidently a reservoir of xerotolerant and xerophilic fungi, which survives to the thermal treatment used to make honey non-crystallisable. Some of these fungi are related to the honeybee life-style; however, as is in the case of the new taxa described here, the origin in nature remains unknown. In the latter case, flowers and aphids could play an important role as a source of such fungi. During the course of the study, the most important pathogenic fungi for honeybees, *Aspergillus flavus* and *Ascosphaera apis*, were not found. Several of the fungi found in honey samples (*Aspergillus* and *Pencillium* spp.) are potential producers of mycotoxins, but this does not mean that the honey may represent a risk to the health of the consumer, because (in general) the production of mycotoxins or the fungal growth are suppressed at water activities lower than 0.70 (Mannaa & Kim 2017), as is the case of honey (a_w_ of 0.60 or less). Honey should be considered as a “living food” and, consequently, its “normal” mycobiota merits more extensive study. It is expected that such “normal” mycobiota may vary qualitatively and quantitatively, depending on the geographic origin, the botanical type and water activity of the honey, among other physicochemical and biological parameters. Honey is clearly one of the relatively unexplored habitats for the missing fungal diversity, especially as the new taxa we found came from samples from just two countries.

## Data Availability

All data generated or analyzed during this study are included in this published article.

## Abbrevations

a_w_: water activity
BCP-MS-G: Bromcresol purple milk solids glucose agar
BEA: Bile esculin agar
*BenA*: fragment of the beta-tubulin gene
BI: Bayesian-inference
BLAST: Basic Local Alignment Search Tool
*CaM*: fragment of the calmodulin gene
CREA: Creatine sucrose agar
CYA: Czapek yeast extract agar
DG18: Dichloran 18% glycerol agar
DNA: Deoxyribonucleic acid
G18 DG18: without dichloran
G25 N 25%: glycerol nitrate agar
ITS: Ribosomal internal transcribed spacers
LSU: Large sub unit of the ribosomal genes
MEA: Malt extract agar
ML: Maximum-likelihood
MLI: Maximum level of identity
MY70FG: Malt extract yeast extract 70% fructose-glucose
nrRNA: Nuclear ribosomal ribonucleic acid
OA: Oatmeal agar
PDA: Potato dextrose agar
PYE: Phytone yeast extract agar
*rpb*2: fragment of the RNA polymerase II subunit 2 gene
SEM: Scanning electron microscopy
TOTM: Test opacity tween medium
TreeBASE: a repository of user-submitted phylogenetic trees and data used to build them
YES: Yeast extract sucrose agar
